# Nursing Process in Primary Care: perception of nurses

**DOI:** 10.1590/0034-7167-2020-1109

**Published:** 2022-06-24

**Authors:** Marta Patricia Spazapan, Dalvani Marques, Beatriz Pera de Almeida-Hamasaki, Elenice Valentim Carmona

**Affiliations:** I Universidade Estadual de Campinas. Campinas, São Paulo, Brazil.

**Keywords:** Nursing Process, Primary Health Care, Community Health Nursing, Public Health Nursing, Qualitative Research, Proceso de Enfermería, Atención Primaria de Salud, Enfermería en Salud Comunitaria, Enfermería en Salud Pública, Investigación Cualitativa, Processo de Enfermagem, Atenção Primária à Saúde, Enfermagem em Saúde Comunitária, Enfermagem em Saúde Pública, Pesquisa Qualitativa

## Abstract

**Objectives:**

to understand the perception of Primary Health Care nurses about the application of the Nursing Process.

**Methods:**

this is a qualitative, descriptive study. Data was collected through semi-structured interviews with Primary Health Care nurses from a city in the interior of the state of São Paulo, analyzed by Content Analysis under the theoretical framework of Work Process.

**Results:**

three categories were obtained: Extrinsic factors to the Nursing Process; Intrinsic factors to the Nursing Process; and Knowledge.

**Final Considerations:**

the nurses considered the Nursing Process relevant for the profession, but historical, political, and social issues related to nursing, and health, as well as conflicts regarding its concept and academic training, hinder its application.

## RESUMO

**Objetivos:**

compreender a percepção dos enfermeiros da Atenção Primária à Saúde sobre a aplicação do Processo de Enfermagem.

**Métodos:**

estudo qualitativo, descritivo. Os dados foram coletados por entrevistas semiestruturadas com enfermeiros da Atenção Primária à Saúde de uma cidade do interior do estado de São Paulo, analisados pela Análise de Conteúdo sob o referencial teórico de Processo de Trabalho.

**Resultados:**

obtiveram-se três categorias: Fatores extrínsecos ao Processo de Enfermagem; Fatores intrínsecos ao Processo de Enfermagem; e Saber.

**Considerações Finais:**

os enfermeiros consideraram o Processo de Enfermagem relevante para a profissão, mas questões de ordem histórica, política e social relativas à enfermagem e à saúde, bem como conflitos quanto ao seu conceito e à formação acadêmica, dificultam sua aplicação.

## RESUMEN

**Objetivos:**

comprender la percepción de los enfermeros de la Atención Primaria de Salud sobre la aplicación del proceso de enfermería.

**Métodos:**

estudio cualitativo, descriptivo. Los datos fueron recolectados por entrevistas semiestructuradas con enfermeros de la Atención Primaria de Salud de una ciudad del interior del estado de São Paulo, analizados por el Análisis de Contenido bajo el referencial teórico de Proceso de Trabajo.

**Resultados:**

obtenidos tres categorías: Factores extrínsecos al proceso de enfermería; Factores intrínsecos al proceso de enfermería; y Saber.

**Consideraciones Finales:**

los enfermeros consideraron el proceso de enfermería relevante para la profesión, pero cuestiones de orden histórico, político y social relativas a la enfermería y a la salud, así como conflictos cuanto a su concepto y a la formación académica, dificultan su aplicación.

## INTRODUCTION

This study was initiated based on concerns regarding the notifications about the Nursing Process (NP), arising from the inspection of the Regional Council of Nursing (COREN) - Campinas subsection to the health units of the Municipal Health Secretariat (SMS) of the Municipal Government of Campinas (PMC). It was found heterogeneity in the registration of the NP by nurses of the Primary Health Care (PHC). This lack of homogeneity in the application of the NP in PHC is not a reality limited to nurses of the aforementioned context, since factors such as the preparation of the future professional and the level of incentive from institutional organizations for the application of the NP can interfere in its realization^(1,2,3)^.

The use of the NP contributes to the effectiveness of the nurse’s work in PHC, obtaining better results with the assisted population^(4)^. However, in clinical practice, there are limitations in the implementation of the NP, in Brazil and in other countries, with failures in the records of one or more of its phases^(3,4,5,6)^. In addition, the experiences of improvement of the NP documentation in Brazil still prevail in the hospital environment, in secondary and tertiary care levels, as opposed to PHC^(5)^.

The NP is understood as a methodological tool that requires cognitive, technical, and interpersonal skills, since it must be developed and executed according to the needs of the person, family, and/or community that demand professional care, focusing on problem-solving in a deliberate way^(7)^.

In this study, Nursing Care Systematization (NCS) and NP are understood as distinct: the NP is a methodological instrument that directs care and organizes documentation; while NCS organizes professional work in terms of method, people and instruments, making it possible to implement the NP^(8)^. This distinction is relevant because the conflicting definitions of terms related to the NP can also be considered a factor that hinders its understanding and consequent application in professional practice^(9-10)^.

In addition to its conceptual aspects, the NP is an integral part of legislation that provides legal support to the nursing profession and describes the private activities of nurses^(11-12)^. Among them, COFEN Resolution 358/2009 deals with the NCS and implementation of the NP in all instances where there is nursing care, reaffirming the NCS as the organizer of the work and the NP as a methodological instrument. Finally, COFEN Resolution 358/2009 and Resolution 429/2012 deal with the records of all care provided and the NP^(8,13)^.

It is understood that the application of the NP is mandatory in all contexts where nurses work with the individual, family, and community. PHC is inserted in the Brazilian Unified Health System (UHS) as a strategy to organize the healthcare model in order to respond to most of the health needs in a regionalized, continuous, and systematized way, combining preventive and curative actions, as well as care for individuals and communities^(14)^.

Over the decades, nursing has built relevant practices in PHC, which contributed to the establishment of the UHS. From managerial to care and/or educational activities, nurses have diversified and expanded the scope of care that they perform, in order to ensure care to the human being in all stages of life. The organization of PHC in the country with the Family Health Strategy has favored and encouraged the incorporation of different organizational arrangements, stimulating the use of various technologies, especially relational ones such as bonding and qualified listening^(15)^.

Faced with so many actions and technological advances, the NP aims to positively interfere in the assistance^(16)^, since it organizes and directs clinical reasoning and the provision of individualized nursing care, aiming at higher quality. Besides the fact that the NP has legislation in the Council of Class, it has recognized value by directing the record of information that is exchanged between nurses and patients, related to the routing of effective care. Thus, the development of a study on the perceptions of PHC nurses regarding the NP can shed light on the understanding of the low adherence of this in clinical practice and raise future strategies to change this scenario.

## OBJECTIVES

To understand the perception of Primary Health Care nurses about the application of the Nursing Process.

## METHODS

### Ethical Aspects

This study respected the ethical aspects as recommended by Resolution 466/12 of the National Health Council, and was approved by the Research Ethics Committee of the State University of Campinas (UNICAMP). All nurses were instructed about the objectives and procedures of the study, read and signed the Free and Informed Consent Term (FICT).

### Theoretical framework

The theoretical reference of the Work Process was used, which understands that the individual, when performing his activity, transforms an object, using instruments that enable the elaboration of products. This process defines some elements such as the agent, the object of work, the instruments, and the purpose of this work. In Health, work is understood as a social practice, articulated to the social and historical context, being collective^(17-18)^.

The process of health work happens when it is performed, that is, it is in the act. To carry out this work, professionals use a set of instruments/technologies, namely, hard technologies (equipment, structures), soft technologies (technical knowledge, know-how), and soft technologies (everything that is established in the relationship between the professional and the user)^(19)^. Therefore, the NP was delimited as a soft technology in the context of the Work Process.

### Type of study

This is a qualitative study of the descriptive type, which is defined as reflexive and interpretive, because it makes it possible to consider a range of perspectives of the participants, with identification of variables that could not be measured, listening to the other, and minimizing power relations^(20-21)^. The Consolidated Criteria for Reporting Qualitative Research (COREQ) was adopted to guide the methodology.

### Study Scenario

The study setting was the Eastern Health District of the city of Campinas, State of São Paulo, Brazil. It has ten Basic Health Units with 30 nurses, as well as Health Surveillance, Psychosocial Care Center (PSCC) 3, PSCC Alcohol and Drugs, Children’s PSCC, Home Care Service (HCS), and Specialty Outpatient Clinic. Its population was 246,866 inhabitants, distributed in more than 300 neighborhoods. In the city in question, the Health Care Network is complex and districtized, with Primary Care services (64 Health Centers), specialized services, diagnostic support, urgency, and emergency care, and hospital care, distributed in five health districts, with geographical areas of approximately 200 thousand inhabitants each.

### Data source

The subjects of the study were nurses from the mentioned health district. The sample was intentional because it was a setting in which there had not yet been systematized discussions about NP, as held in other health districts in the Primary Care network. Nurses in management positions and those who were away on vacation or leave at the time of data collection were excluded.

### Data collection

Data was collected through a semi-structured interview, whose script contained two parts: the first part referred to the collection of data on subject characterization, and the second part contained the triggering statement and the guiding questions. The initial contacts between the first author and the nurses were made by email, telephone contact, and/or electronic messaging system (WhatsApp), from January to February 2017. The interviews took place in February and March 2017. The instrument was tested before its use in the interviews, with three people who were not part of the sample: two nurses and a graduate student. The interview began with the triggering statement: “Tell me about your experience with the Nursing Process in Primary Care”. Two guiding questions were also introduced: “How do you perceive the Nursing Process for a professional qualification?” and “How would you define Nursing Process?” The interviews were audio-recorded, and were transcribed in full.

### Data analysis

The transcribed material was submitted to Content Analysis: this is a way of revealing the “nuclei of meaning” that are elements of communication and whose presence may mean something to the object that one intends to analyze. The data analysis was carried out in the following stages: pre-analysis; exploration of the material and treatment of the results obtained; and interpretation^(21)^.

## RESULTS

### Nurse’s profile

Twelve nurses were interviewed, with ages ranging from 30 to 50 years old. Regarding postgraduate studies, most of them (9; 75%) had one or more specializations. Of these, six had a post-graduation in Family Health, while two had a post-graduation in Public Health. Thus, it can be seen that most of the subjects sought training to work in PHC. Nine nurses had graduated more than ten years ago. Regarding the time of work in PHC in Campinas, one of the interviewees counted five years, while the rest had more time; of these, six had worked for more than 16 years.

Considering the speeches, three categories emerged: “Extrinsic factors to the Nursing Process”, “Intrinsic factors to the Nursing Process”; and “Knowledge”. They are presented below with the speeches that represented them.

### Extrinsic factors to the Nursing Process

The participants’ statements refer to the historical, social, economic, and structural issues of the institution or workplace, evaluated as negative interferences to the application of the NP. They also reported that the lack of physical structure of the workplace hinders the nurse’s performance and, consequently, the implementation of the NP:

[…] *first, because we are living, ah* [...] *a difficult situation in the country: this economic crisis. So, all this has an aggravating factor, a direct impact for Primary Care as the gateway to the system.* (N5)

*Another thing that does not help, but I am policing myself more, is the lack of structure, so* [...] *to do the reception I am not always in a room there* [...] *and organized to do that. Sometimes, I look at a patient here and there* [...] *and then I leave the report to do later, to attend the next patient. Then when I see it, it’s lost* [...] *then later* [...]*. What I think makes it difficult is the structure and the time. I think that maybe because it is not instituted in our culture. We don’t like to let the patient wait and accumulate files and that’s it.* (N8)

In the report below, the issue of the work process is evidenced and the historicity of nursing, its relations with other professions and institutions are pointed out:

*So, like this, I couldn’t open a Pap smear schedule. I have no physical space for the demand of work.* [...] *we don’t do* [the exam] *because we have four gynecologists and we don’t have a room because the University* [cites the name of a University] *attends all the time here. If they are not here, the gynecologists are here. So, we don’t have a room.* [...] *They are going to reform* [...]*. But we were not called to discuss how this reform will be done: if it will be just a makeover or if, suddenly, they will build another room, if they will improve the physical space.* (N11)

The excess and diversity of tasks and actions in nursing practice appear in the speeches as factors that interfere with the work process:

[…] *what I observe is the multitasking or multi-tasking that the nurse does here. The nurse becomes the leader of a team that is not only a nursing team. And we are accessed all the time, regardless of what we are doing, you know? So, we are accessed for emergencies, we are accessed for administrative issues, we are accessed for surveillance issues that do not belong in that environment, I mean, at that moment, really. So, I understand that the first thing is the multitasking that leaves us, within the consultation, being interrupted all the time* […]. (N12)

[…] *At least, for doctors, I think it’s easier because they have that amount of patients scheduled and that amount of reception per day* [...]*. They have a limit. And nursing works with a totally open door. You have to deal with this demand that comes knocking at our door and then it’s* [...] *it’s very stressful the way we are working.* (N5)

The reception was also frequently mentioned as an external factor related to the care model in force in the country, which can interfere with the NP. They also mention the way the work is organized inside the unit: the fact that the way the reception is performed in the units of the studied municipality is not uniform:

[…] *the issue of reception* [...] *of being forced to go through* [...] *being forced to do* [...] *they do it in a way that we have no leg to stand on* [...]*. The demand swallows* [the nurse]. (N4)

The speeches directed to COREN describe it as an organ related to inspection:

*Well, now it’s even improved a little bit* [...] *but so* [...] *before, so... we didn’t write almost anything* [...] *the notes were very poor* [*..*.]*. So, we started to show it, right?* [...] *COREN even came here and saw the need to write, to identify oneself, right? To stamp and sign what the professional is doing, because many times a person would come to check their BP* [blood pressure]*, a basic example* [...] *ten times a year, and not once would it be written down. So, this is a document, right? So, the notes are much better* […]. (N3)

### Intrinsic factors of the Nursing Process

The speech below represents the perception of the interviewees that there is a distancing of the NP in relation to PHC:

*I think that it* [the NP] [...] *in primary care it is kind of* [...] *how should I say* [...] *inside the hospital it is easier for us to apply because you are seeing the patient all day* [...]*. So, we don’t have much incentive to be doing all the steps as it would be in the hospital. There you see the patient every day* [...] *you see if he is evolving or not, it is easy to follow up, but inside the Basic Unit, it is very difficult.* (N1)

However, nurses also discuss the peculiarities of the NP in PHC, especially considering the periodicity of the user’s return, which is very variable:

*In relation to the total application of the NP in the health center* [...]*. Because we can make an evaluation of the subject in the scheduled appointments, so, in individual care, we can* [...] *eh* [...] *do an anamnesis, right? Finding problems to intervene, making specific interventions, but we can’t make a periodic evaluation of this subject, for example, unless it’s a very serious case that comes back weekly* […]. (N12)

*The process is the whole, all the steps that we do, systematization is the sense of organizing the process as a whole and recording it, so systematization in this sense of the dictionary, of organizing the Nursing Process that we perform and recording it* [...]*. Nursing record: we fail in the record of the process as a whole.* (N9)

The speeches also portray confusion between the concepts of the NP and the NCS, in addition to the lack of approximation with the legislation in force:

[…] *Nursing Process is all the team readjustment, all the restructuring* [...] *that I realize* [...] *we elaborate all the processes, eh* [...] *room scales, eh* [...] *how can I say* [...] *procedures and everything else.* (N2)

[…] [what the NP is about] *routines, tidying up the rooms, each one in his function* [...]*. It is good to apply NCS. We don’t apply it, because there is no way, right?* [...] *Except for bedridden patients, when we go for home visits* […]. (N3)

### Knowledge

Nurses bring the influence of knowledge in the application of the NP, which they highlight both in their own training and in the training of undergraduate nursing students with whom they have contact:

*Look, to be very honest, I didn’t have good training in this part of the Nursing Process. Today as a professional, I see how bad it was. I applied more Nursing Processes in the hospital. Eh, maybe not the way I understand it today, in a health center. In college, if I did two - oh, no, not even two - it was a lot. Not good training. Then I discovered the CIPESC, right here in the city hall, in a meeting or another* […]. (N8)

*Now I’m with a student here and I also realize that even the nursing student doesn’t know. And I say, “Can’t you talk about?” And they* [answer], *“I don’t know either.”* (N11)

However, the nurses also point to the NP as a qualifier of professional practice:

*So what I said* [...]*. I think it is super good for a professional qualification, what I said. He* [the professional] *ends up having much more autonomy because we will be qualifying him for this. I think that the process* [Nursing Process] *qualifies the professional, and then he will have more autonomy to do his part. That’s it!* (N4)

## DISCUSSION

It appears in the speeches of professionals the problems of work organization in PHC, such as lack of structure, are related to the difficulties of investment in public health in Brazil and the gradual dismantling of the UHS, both nationally and municipally. This perception is strengthened by the fact that the sample is composed of nurses with experience in PHC and trained to do so, following the national and state trend^(22)^.

Nursing is social work, since it occurs in relationships with other professions and in various institutional and social spaces. Thus, nurses work in a multi-professional and multifaceted context, and their work must be connected to the whole, in a context in which there is a range of subjects, actions, attributions, legislation, management, organizations and technologies, financial resources, and their application modalities. In this scenario, we find nurses and their work; according to the speeches, they need to cope with the demand, the demands inherent to the profession inside and outside the health unit.

The change in the healthcare model, with the proposal of multi-professional care to users, has not yet caused the offices to stop being spaces of privilege, where the medical professional has his established space of power. Managers, both in the studied units and in other spheres, have not managed to incorporate the understanding that other players have their own and autonomous knowledge and can also act through consultations with the user. This perspective can be explained by the fact that it is considered that the physician is in charge of intellectual actions in the relationship with the user, while nursing is in charge of manual actions, procedures, and does not need a specific space for its performance^(18)^.

The interviewees mentioned the need for nurses to have a voice in decision-making processes related to the work process and the structure of the unit. They show discontent for not feeling considered in decisions and not having the physical space for the performance they desire. It is noted that the movement in the work of nurses in PHC is empowering and contradictory, full of subjectivities, causing subjects to identify themselves sometimes as protagonists in the practices they perform, sometimes as assistants in the work of other professionals^(18)^. These findings reinforce health work as living work, performed in the act^(19)^.

Overwork is a factor cited by nurses as something that hinders the application of the NP^(23,24,25)^. Associated with this is the way services and primary care are organized, which overloads this professional category^(24-25)^. Overwork and the multiplicity of nurses’ actions have been addressed by studies in different periods, continuing to be a reality, which shows that this scenario, both in managerial and care aspects, interferes negatively in the nurse’s work and, consequently, in the application of the NP^(3,4,5,9,14,15,16,24-25)^. In addition, such a situation reduces patient safety and nurses’ satisfaction with their work process.

One aspect that draws a lot of attention is the statements about legislation since the nurses only mention and describe the COREN’s visits to the units, not addressing the specific issue that the NP is part of the legal apparatus of the profession. Thus, they do not present in their statements the relevance of records for the professional’s clinical reasoning process, nor do they discuss the legal aspects of these records in health care. They do not even mention the NP as a work method that consecrates nursing as an established profession, as something that raises its scientificity.

The interviewees demonstrate the need for empowerment, despite nursing having a long history in public health and an important insertion in PHC since there has not yet been the incorporation of professional autonomy processes in the way that could and should be^(14,24)^. It can be understood that nurses have not appropriated the NP as a technological tool, that is, they do not recognize it as an organized way of expressing their scientific knowledge, perceiving it only as something related to a set of regulations with which they have little approximation.

These same nurses claim that, in the hospital environment, patient care is more predictable and controlled, and this leads them to believe that, there, the NP is better performed and easier to apply than in PHC. Thus, they disregard that the hospital nurse can also perform NP for patients that he/she does not fully know or accompany on a daily basis. The unfamiliarity with each patient only emphasizes the value of NP-related records, regardless of the care setting. In addition, the NP in PHC could be used to investigate the frequency of return for some specific cases, while contemplating the individualization of care. This instrument would allow the characterization of customers regarding the most prevalent nursing phenomena and would enable a database, which would facilitate the identification of interventions that were effective or not.

Confusion between the concepts of NP and NCS was also noted, the reason for which may be the changes in the legislation of the professional practice that have occurred in the last decades, as well as the insertion of new nomenclatures, being NCS used only in Brazil^(8,11,12,13,25)^. The reports expressed fragility regarding the understanding of what is NP, NCS, nursing consultation, and work process. This confusion may be a trigger for the limitations of records and studies on the NP in PHC. Some studies use NP as synonymous with NCS^(5)^, or as a synonym for nursing consulta-tion^(26)^, which confuses, even more, the professionals less used to the concepts and current legislation.

Nurses perceive knowledge about NP as a fundamental element for its application and registration, however, at the same time; they believe it to be deficient since graduation, which implies the incomplete and incorrect use of this technology.

Knowledge is also a technological tool that improves nurses’ actions in care. Nurses sometimes claim to use this knowledge, but say they have difficulty in recording the action; sometimes they say they do not record it, so they claim not to perform the NP. Therefore, the failure to register may raise a mistaken perception of the non-existence of the application of the NP. Regarding the application in PHC, they also point to unpreparedness since their academic training, and this corroborates findings from other studies^(5,24-25,27-28)^.

The reports seem to reveal the desire both to learn more about the NP and to apply it as a tool to qualify the professional practice, which is relevant. The literature points out that teaching provides an increased use of this technology and improved records^(2-3,25,26,27,28)^. Thus, in-service education activities can have a positive impact in this context.

As highlighted by the nurses’ comments, the Nursing Process is influenced by factors extrinsic to its use in PHC, which permeates the logic of service organization, the institutional structure, and the way nurses’ work is defined in this scenario. As a technology proper to the nursing profession, the NP is not effectively used or recognized as knowledge, know-how, a part of the nurses’ daily work. Thus, it is considered idealized and not feasible to be incorporated into PHC practices. Therefore, it is urgent to provide support to the team to understand this instrument beyond such perception, and this supply can be given through in-service education and working conditions to implement it in a satisfactory, enjoyable, and meaningful way for nurses.

Thus, as subjects, agents, nurses experience macro-political issues, care model, structure, and professional training that interfere in their own work, causing them not to recognize the NP as their own knowledge, a technology that would enhance their practices as affirmative actions in the work performed with users, families, and community, as well as local health teams. Such recognition would allow the NP to be implemented in a more intentional way to guide actions in the micro-politics of the teams’ work processes, giving new meaning to what they do and why they do it.

Intervention studies should be developed to overcome barriers to the implementation of the NP at this level of care. The dissemination of successful experiences can also help this process in units that experience difficulties. In other countries, the NP and the application of standardized nursing language are better established as to implementation, reaching the level that data from them are used as predictors of patient complexity and mortality, workload, and costs related to care^(29,30)^. However, these studies are also still more frequent in the hospital setting.

### Study limitations

The interviews with nurses from only one of the five Health Districts of Campinas can be considered a limitation. However, this district was chosen because of peculiarities such as its extensive coverage area and the lack of participation in recent continuing education on NP at the time of data collection of the study.

### Contributions to nursing

This study contributes to nursing as a profession, as it offers subsidies to think about strategies to improve the application of the NP in PHC and the consequent improvement in the recording of the actions developed by nurses in this setting. It is suggested a better approximation of legislation with the practice of nurses in PHC; understanding of the objectives of work in PHC by the legislators of nursing in order to demystify the application of the NP in this context; creation of strategies that enable PHC professionals to appropriate the concepts of NP and NCS; and reformulation of the teaching of the NP in the undergraduate course in order to reduce distortions in its application in daily practice. That said, this work contributes to the health of the population because it qualifies the assistance provided to the community.

## FINAL CONSIDERATIONS

When seeking to understand the perception of Primary Health Care nurses about the application of the NP, it was found that their speeches bring situations that are not specific to such application, but that compromise it. The implementation of the NP is linked to the historicity of nursing work, its social relations, as well as its interactions with other professions, especially with the medical professional, with whom it still maintains a power struggle for institutional space.

As for the specific situations of the NP that interfere in its implementation, nurses see some unfeasibility of using this instrument in PHC and incompatibility with their professional duties in this space. The work of nurses in PHC was built with a focus on other knowledge, and the NP is perceived as more easily applicable to the hospital context. Moreover, it became evident the difficulty of nurses to adequately conceptualize what is NP.

Thus, there are challenges to the implementation of the NP in the context of PHC, such as: the nurses’ knowledge of the current legislation for professional practice; the reconfiguration of the nurses’ training in NP during graduation in order to enable them to apply it in different contexts; the effectiveness of continuing education on the NP, differentiating it from the NCS, for its incorporation into the nurses’ daily practice as an instrument that enhances and gives visibility to the work of these professionals.

## SUPPLEMENTARY MATERIAL

The master’s thesis that originated this article can be accessed via the Brazilian Digital Library of Theses and Dissertations by accessing the link http://repositorio.unicamp.br/.

0034-7167-reben-75-06-e20201109-sup01
