# Supplementary material for: Nursing Process in Primary Care: perception of nurses
Source: Rev Bras Enferm. 2022 Jun 24;75(6):e20201109. doi: 10.1590/0034-7167-2020-1109 (PMC9728832; doi:10.1590/0034-7167-2020-1109)
Supplement: 0034-7167-reben-75-06-e20201109-sup01 [file 0034-7167-reben-75-06-e20201109-sup01.pdf]

UNICAMP

UNIVERSIDADE ESTADUAL DE CAMPINAS
FACULDADE DE ENFERMAGEM

MARTA PATRíCIA SPAZAPAN

PROCESSO DE ENFERMAGEM NA ATENÇÃO PRIMÁRIA: PERCEPÇÃO DE ENFERMEIROS DE CAMPINAS-SP

CAMPINAS

2017

MARTA PATRíCIA SPAZAPAN

PROCESSO DE ENFERMAGEM EM ATENÇÃO PRIMÁRIA: PERCEPÇÃO DE ENFERMEIROS DE CAMPINAS – SP

Dissertação apresentada à Faculdade de Enfermagem da Universidade Estadual de Campinas como parte dos requisitos exigidos para a obtenção do título de Mestra em Ciências da Saúde, na Área de Concentração: Enfermagem e Trabalho

ORIENTADOR: Profª. Drª. Elenice Valentim Carmona

ESTE EXEMPLAR CORRESPONDE À VERSÃO
FINAL DA DISSERTAÇÃO DEFENDIDA PELO
ALUNO MARTA PATRíCIA SPAZAPAN, E ORIENTADO PELO
Prof.ª. DR.ª. ELENICE VALENTIM CARMONA.

CAMPINAS
2017

Agência(s) de fomento e nº(s) de processo(s): Não se aplica.

Ficha catalográfica
Universidade Estadual de Campinas
Biblioteca da Faculdade de Ciências Médicas
Ana Paula de Morais e Oliveira - CRB 8/8985

Spazapan, Marta Patrícia, 1971-
Sp29p Processo de enfermagem na atenção primária : percepção de enfermeiros de Campinas-SP / Marta Patrícia Spazapan. – Campinas, SP : [s.n.], 2017.

Orientador: Elenice Valentim Carmona.
Dissertação (mestrado) – Universidade Estadual de Campinas, Faculdade de Enfermagem.

1. Processo de enfermagem. 2. Atenção primária. 3. Enfermagem em saúde comunitária. 4. Pesquisa qualitativa. I. Carmona, Elenice Valentim,1976- . II. Universidade Estadual de Campinas. Faculdade de Enfermagem. III. Título.

Informações para Biblioteca Digital

Título em outro idioma: Nursing process in primary care : perception of nurses from Campinas-SP
Palavras-chave em inglês:
Nursing process
Primary health care
Community health nursing
Qualitative research
Área de concentração: Enfermagem e Trabalho
Titulação: Mestra em Ciências da Saúde **Banca examinadora:**
Elenice Valentim Carmona [Orientador]
Cândida Caniçali Primo
Erika Christiane Marocco Duran
Data de defesa: 06-07-2017
Programa de Pós-Graduação: Enfermagem

BANCA EXAMINADORA DA DEFESA DE MESTRADO

MARTA PATRÍCIA SPAZAPAN

ORIENTADOR: PROFª DRª ELENICE VALENTIM CARMONA

MEMBROS:

1. PROFª DRª ELENICE VALENTIM CARMONA

2. PROFª. DRª. CÂNDIDA CANIÇALI PRIMO

3. PROFª. DRª. ERIKA CHRISTIANE MAROCCO DURAN

Programa de Pós-Graduação em Enfermagem da Faculdade de Enfermagem da Universidade Estadual de Campinas.

A ata de defesa com as respectivas assinaturas dos membros da banca examinadora encontra-se no processo de vida acadêmica do aluno.

Data: DATA DA DEFESA [06/07/2017]

DEDICATÓRIA

Dedico esta pesquisa a todos os enfermeiros, em especial aos da Atenção Primária à Saúde, pois vivem no trabalho diário as dores e as delícias de suas comunidades.

AGRADECIMENTOS

Quando parei para escrever os agradecimentos, me dei conta de que tenho muito a agradecer e isso me encheu de um acalento e uma doçura que não consegui descrever...
Vou começar pela minha família, italiana e barulhenta, que me deu as primeiras noções de amorosidade, que me acolheu na infância, aguentou na adolescência e cuidou nos momentos sombrios da maturidade e ainda está aí para tudo.
Agradeço aos meus pais por me despertarem o gosto pela leitura e pelo aprender sobre as coisas e a vida.
Ao meu pai por ter me ensinado o conceito de ser cidadã e as responsabilidades sociais que vem com esse ensinamento.
À minha mãe que foi meu chão, que odiava viajar ou mesmo mudar de casa, mas me ensinou a voar para onde eu quisesse.
Agradeço a todos os meus professores, desde os que me ensinaram as primeiras letras até os que me deram a bagagem para trilhar os caminhos da profissão que escolhi exercer.
Aos colegas de trabalho que me ajudam diariamente a ser a profissional que sou.
Agradeço aos alunos que me dão o frescor do aprendizado contínuo, que me proporcionam ser jovem por meio deles e que me dão muito orgulho sempre.
À minha orientadora muito especial, amiga de infância, pela paciência e valiosos ensinamentos.
À Dalvani que me incentivou a abraçar o desafio de voltar à academia depois de tanto tempo e ao apoio dado nessa jornada.
Aos Membros da Banca Examinadora pelas contribuições essenciais à qualidade desse trabalho.
Aos meus amigos que são a minha família do coração, que me permitiram rir, chorar, tomar alguns porres, amar, sofrer, se erguer e continuar a caminhar juntos.
Enfim, meu profundo amor e agradecimento a todos os que são testemunhas da minha vida pois, cada qual, com sua peculiaridade moldou aquilo que sou.

RESUMO

O Processo de Enfermagem (PE) é um instrumento metodológico que orienta o cuidado profissional e a documentação da prática, aumentando a visibilidade e o reconhecimento da profissão. Este estudo teve como objetivo compreender a percepção de enfermeiros a respeito do Processo de Enfermagem na Atenção Primária da Secretaria Municipal de Saúde de Campinas e descrever os fatores dificultadores e facilitadores na aplicação do PE na APS. Trata-se de uma pesquisa qualitativa, que utilizou o referencial teórico do Processo de Trabalho. Foram sujeitos do estudo doze enfermeiros que atuavam em Unidades Básicas de Saúde do Distrito Leste de Campinas. Os dados foram coletados por meio de entrevista semiestruturada, em encontro único, gravada em áudio nas unidades de saúde onde os respectivos entrevistados atuavam. Os dados foram analisados segundo Análise de Conteúdo sob a perspectiva do referencial teórico de Processo de Trabalho. Dos discursos dos enfermeiros foram apreendidas três categorias. A primeira delas foi intitulada "Situações extrínsecas que interferem na realização do PE": aqui os relatos representam percepções dos enfermeiros sobre a situação política e econômica, considerando diferentes contextos da gestão que interferem nas condições de trabalho, dificultando a aplicação do PE. A segunda categoria, nomeada "Situações intrínsecas que interferem na realização do PE", apresenta relatos sobre as dificuldades com os passos do processo em si e sua aplicabilidade na Atenção Primária. Por último, a categoria "O saber" apresenta a percepção dos profissionais quanto ao desconhecimento do PE, o que se relaciona a desatualização sobre mudanças na legislação, pouca exploração do assunto na graduação e na atenção primária. Depreende-se que as condições de trabalho dificultam a implementação do PE, visto que os enfermeiros identificam inúmeros obstáculos em seu contexto de trabalho.

Palavras-chaves: Processos de Enfermagem, Atenção Primária, Enfermagem em Saúde Comunitária, Pesquisa Qualitativa.

Linha de Pesquisa: Processo de Cuidar em Enfermagem.

ABSTRACT

The Nursing Process (NP) is a methodological tool that guides professional care and practice documentation, improving nursing visibility and recognition. This study aimed to understand nurses´ perception regarding Nursing Process in Primary Health Care in the Municipal Health Department of Campinas, São Paulo, Brazil. It is a qualitative research based on Work Process theoretical reference. Nurses working at Basic Health Units of the Eastern District of Campinas were subjects of this study. Data were collected through a semi-structured interview, in a single meeting, recorded in audio at the health units where the respective interviewed act. The data were analyzed, from the perspective of the theoretical reference Work Process. Considering nurses' speech, there were three categories. The first one of them was entitled "Extrinsic situations that interfere in the accomplishment of the NP": here reports represent nurses' perceptions about political and economic situations, considering various management contexts that interfere in working conditions, making it difficult to apply the NP. The second category, named "Intrinsic situations that interfere in the accomplishment of the NP", presents reports about difficulties upon steps of the process itself and its applicability in Primary Care. Finally, the subcategory "Knowledge" presents the professionals' perception regarding the lack of knowledge on NP, which is related to shortage of updating about changes in legislation, little exploration of the subject during undergraduate course and at primary care. It can be seen that working conditions hamper implementation of the NP, as nurses identify numerous obstacles in their context.

Keywords: Nursing Process, Primary Health Care, Community Health Nursing, Qualitative Research

LISTA DE ILUSTRAÇÕES

Figura 1. Mapa do Município de Campinas por divisão em Distritos de Saúde..p29

LISTA DE TABELAS E QUADROS

Tabela 1. Distribuição das características dos enfermeiros entrevistados. Campinas – SP, 2017..p41

LISTA DE ABREVIATURAS E SIGLAS

CAPS - Centro de Atenção Psicossocial
CE – Consulta de Enfermagem
CEP – Comitê de Ética em Pesquisa
CIAP - Classificação Internacional da Atenção Primária
CIPE – Classificação Internacional para a Práticas de Enfermagem
COFEN - Conselho Federal de Enfermagem
COREN - Conselho Regional de Enfermagem
EP - Educação Permanente
NANDA-I - NANDA Internacional, Inc.
PE - Processo de Enfermagem
PMC - Prefeitura Municipal de Campinas
POP - Procedimento Operacional Padrão
SAD - Serviço de Atenção Domiciliar
SAE - Sistematização da Assistência de Enfermagem
SMS - Secretaria Municipal de Saúde
RMC - Região Metropolitana de Campinas
TCLE - Termo de Consentimento Livre e Esclarecido
UBS - Unidade Básica de Saúde
UNICAMP - Universidade Estadual de Campinas

Sumário

1.INTRODUÇÃO 13
1.1.A pesquisadora 13
1.2. O contexto da Atenção Primária à Saúde e o Processo de Enfermagem 14
1.3. Processo de Enfermagem: contexto histórico, definição e etapas 16
1.4. Processo de Enfermagem como legislação 20
1.5. Atenção Primária à Saúde 21
2.OBJETIVOS 25
2.1. Objetivos Gerais 25
3.PERCURSO METODOLÓGICO 26
3.1. Cenário de Estudo 26
3.2. Sujeitos do Estudo 29
3.3. Instrumento e procedimentos de coleta de dados 30
3.4. Considerações éticas 32
3.5. Processamento e Análise dos Dados 32
4.REFERENCIAL TEÓRICO 34
4.1.O Processo de Trabalho em Saúde 34
4.2.Processo de Trabalho e a Enfermagem 36
4.3.Organização Tecnológica do Trabalho em Saúde 38
5. RESULTADOS E DISCUSSÃO 40
5.1. Caracterização dos participantes 40
5.2. Os relatos dos enfermeiros e as categorias temáticas 43
5.2.1 Situações extrínsecas que interferem na realização do PE. 43
5.2.2 Situações intrínsecas que interferem na realização do PE. 52
5.2.3 O Saber do Enfermeiro 56
6. CONCLUSÃO 60
7. REFERÊNCIAS 63
8. APÊNDICES 69
Apêndice 1 69
Apêndice 2 70
Anexo A 73
Anexo B 74

1.INTRODUÇÃO

1.1.A pesquisadora

Por se tratar de uma pesquisa de cunho qualitativo, falar dos sujeitos envolvidos nela se faz premente. Assim inicio falando do sujeito que deflagra este estudo, no caso a autora, que sou eu.

Sou enfermeira há 20 anos, todos eles na Secretaria de Saúde de Campinas, dos quais 15 dedicados à Atenção Primaria à Saúde (APS), cuja paixão nasceu ainda na graduação e que foi paulatinamente alimentada com os anos de trabalho. Os períodos fora da assistência direta em Unidade Básica de Saúde (UBS) foram em gestão e em um Pronto Atendimento do mesmo município. Os anos fora da APS me fizeram, mais uma vez, compreender minha vocação como enfermeira sanitarista e assistencial de atenção primária.

Não nasci enfermeira e a paixão pela Saúde Pública e APS se formou por um anseio político, social e de cidadania, forjado no período de franca abertura política nacional, primeiras eleições diretas, primeiros anos de Constituição Cidadã e do Sistema Único de Saúde (SUS). Desta forma minha trajetória na profissão se mistura à trajetória do SUS no Brasil e mais intimamente com o SUS de Campinas-SP, o qual permitiu me tornar a enfermeira que sou hoje.

Nesses 20 anos houve mudanças envolvendo o arcabouço de ações do SUS Nacional e do SUS Campinas bem como mudanças de modelos assistenciais e, consequentemente, na forma como a enfermagem é praticada e como o profissional enfermeiro se insere nesse processo histórico ao qual também me insiro.

Concomitante às referidas mudanças nascem os desafios das políticas públicas, dos modelos assistenciais, das gestões municipais e da enfermagem que, enquanto profissão, também tem seus desafios particulares de consolidação do seu fazer e isso inclui mudanças curriculares, de legislação e maior inserção na pesquisa, bem como em outros espaços.

Todas essas questões suscitam um aprender a fazer contínuo para a construção e consolidação da minha prática enquanto enfermeira na atenção primaria inserida no SUS.

Esses cenários caminham e se misturam, já que não há divisão didática na vida real, ser enfermeiro da atenção primaria e ser enfermeiro.

Assim, ser enfermeira de APS já fazia todo sentido, mas as questões da enfermagem constituída de um corpo legal e inserida na área da saúde era um objeto que ficava distante. Então, em 2013, num desses momentos de aproximação dessas duas facetas da profissão, devido a uma visita do COREN (Conselho Regional de Enfermagem) - subseção Campinas, fui notificada por não realizar Processo de Enfermagem sendo o princípio de vários movimentos que me levou a este estudo.

1.2. O contexto da Atenção Primária à Saúde e o Processo de Enfermagem

Este estudo teve como motivação inquietações frente às notificações sobre Processo de Enfermagem (PE), advindas de visitas realizadas pelo Conselho Regional de Enfermagem (COREN) – subseção Campinas à várias unidades de saúde da Secretaria Municipal de Saúde (SMS) da Prefeitura Municipal de Campinas (PMC) nos anos de 2013 e 2014. Estas inquietações se deram após observar a heterogeneidade na realização do PE por parte dos enfermeiros da atenção primária, bem como na compreensão das definições deste. Por não ter, no momento das visitas técnicas do COREN, uma clareza sobre o assunto a autora deste trabalho também se inclui nesse conjunto de enfermeiros.

Como as visitas do COREN se deram em todos os Distritos de Saúde da SMS, houve uma mobilização dos diversos níveis hierárquicos da Secretaria, no sentido de apropriar-se das definições e conceitos relacionados ao PE e iniciar discussões e capacitações sobre esse tema tão pertinente à prática do cuidado de Enfermagem. Desde então surgiram grupos de discussão que tiveram como produto o desenvolvimento de protocolos, atualização de protocolos anteriores, desenvolvimento do Regimento Interno de Enfermagem e de Procedimentos Operacionais Padrão – POP[1]. Isto com intuito de instrumentalizar enfermeiros e fomentar conhecimento para implantação do PE na atenção primária. Este processo também aconteceu de maneira heterogênea em cada Distrito e Unidade Básica de Saúde (UBS). Ainda assim, mesmo reconhecendo em cada espaço um contexto diferente, faz-se imprescindível salientar que o trabalho iniciado promoveu avanço para a Enfermagem de Campinas e visibilidade da profissão.

A referida cidade possui grande potencial por ser importante polo de universidades e centro industrial e tecnológico[1]. Também oferece campo de estágio para faculdades e cursos técnicos de enfermagem, influenciando o processo de

aprendizagem dos novos profissionais. Atualmente a SMS conta com mais de 300 enfermeiros na atenção primária, número bastante expressivo, se considerarmos este contingente como sujeitos históricos da enfermagem, profissão de um saber técnico e científico estruturado.

A falta de homogeneidade na aplicação do PE na atenção primária não se refere apenas aos enfermeiros da SMS de Campinas, pois muitos fatores interferem na realização do PE, sendo a sua consolidação, dependente de fatores variados destacando-se os econômicos e sociais que antecedem e dão sustentação aos determinantes profissionais[2]. Estas interferências ocorrem mesmo sendo o PE uma parte constante da lei do exercício profissional. Outros fatores significativos que provocam interferência referem-se ao preparo do futuro profissional durante a graduação e as organizações institucionais favoráveis ou não à implementação do PE[3]. Corroborando com esse cenário, um estudo espanhol relata que, apesar dos benefícios da aplicação do PE para a qualidade da assistência, há dificuldades na sua aplicação por motivos semelhantes[4].

A implantação do PE na APS ainda ocorre de forma incipiente e fragmentada, mesmo sendo ele objeto de reflexão desde a década de 70, com os estudos de Wanda Horta[5]. Contudo, ainda que seja um instrumento de grande relevância para a prática da profissão, não é sempre utilizado pela enfermagem brasileira como tecnologia do cuidado[6], apresentando um grande descompasso entre a produção do conhecimento e sua real aplicação na atuação diária do enfermeiro[2].

Na prática clínica existem dificuldades na implantação e utilização do PE, no Brasil e em outros países, com falhas nos registros de uma ou mais fases deste[7;8;9].

Aparentemente, as mudanças de modelo assistencial que vem ocorrendo nas últimas décadas, focando-se na atenção primária e mais precisamente na Saúde da Família e Comunidade, não tem influenciado de forma decisiva a implantação do PE na APS. As experiências de implantação do PE no Brasil são prioritariamente em ambiente hospitalar, nos níveis de atenção secundário e terciário, mantendo-se ainda de forma incipiente na atenção primária[8].

As teses e dissertações desenvolvidas a respeito de PE na atenção primária são em pequeno número[10].

1.3. Processo de Enfermagem: contexto histórico, definição e etapas

PE é um instrumento metodológico que orienta o cuidado profissional de Enfermagem e a documentação da prática, aumentando a visibilidade e o reconhecimento da profissão. Quando realizado em instituições prestadoras de serviços ambulatoriais de saúde, corresponde ao usualmente denominado nesses ambientes como Consulta de Enfermagem (CE)[11].

Para alguns autores a CE está inserida no PE no qual contempla suas etapas[12] ou é proposta como exemplo de aplicação individual do Processo de Enfermagem[13].

Embora essas duas denominações sejam aceitas, será utilizado nesse trabalho a denominação “Processo de Enfermagem” por ser dessa forma que é apresentado pelo COREN em suas abordagens aos enfermeiros no ato das visitas. Além disso a APS possui características mais abrangentes que a atenção ambulatorial, propondo tecnologias e ações mais complexas, sendo exemplos a visita domiciliária, avaliação de curativos, projetos terapêuticos singulares e coletivos, bem como as avaliações da demanda espontânea ou acolhimento. Assim, o conceito Processo de Enfermagem pode ser entendido como algo mais abrangente que o ato individual da consulta, envolvendo a atuação conjunta da equipe cujo o enfermeiro é o norteador do processo de cuidar, utilizando o raciocínio clinico para além das paredes do consultório.

A concepção de PE é antiga e surge com a enfermagem moderna, quando Florence Nightingale acreditava ser necessário que as enfermeiras fossem ensinadas a usar a observação e julgamento critico na sua prática[14]. Porém, foi só na década de 50 que o termo PE foi introduzido na linguagem profissional pelas enfermeiras norte-americanas. Isto porque reconheciam que desenvolver um corpo de conhecimento era vital para a sobrevivência e evolução da profissão, tendo como objetivo dar sustentação científica ao cuidado, com conhecimento próprio, dinâmico e com várias formas de expressão[15].

Sendo o PE desenvolvido e executado tendo em vista as necessidades da pessoa, família e ou coletividade que demandam cuidado profissional para solução de problemas de forma deliberada, exige habilidades cognitivas, técnicas e de relação interpessoal[15].

O desenvolvimento de Teorias de Enfermagem também se deu neste momento histórico e com o mesmo objetivo: dar sustentação teórica e conhecimento próprio à enfermagem[15].

A enfermeira brasileira pioneira em abordar o PE foi Wanda de Aguiar Horta, entre as décadas de 60 e 70: a princípio divulgando o conhecimento das enfermeiras norte-americanas e, posteriormente, divulgando o seu próprio, por meio da Teoria das Necessidades Humanas Básicas, inspiradas em Maslow[15].

No Brasil também é utilizado o termo "Sistematização da Assistência de Enfermagem" (SAE), o que é bastante particular da regulamentação da enfermagem brasileira. Assim, verifica-se que existem algumas discussões e estudos que abordam SAE e PE de formas diversas, além de inúmeras denominações tidas como sinônimos: "Metodologia da Assistência de Enfermagem", " Metodologia da Assistência", "Planejamento da Assistência", "Processo do Cuidado", "Metodologia do Cuidado", "Processo de Assistência", "Consulta de Enfermagem", "Processo de Atenção em Enfermagem" e a própria "Sistematização da Assistência de Enfermagem". Por vezes, as definições são equivocadas. Também há referência de que SAE é uma terminologia que é inadequada ao fenômeno proposto, visto que sistematizar pode significar organizar, mas sem necessariamente usar um método científico[16].

O conflito de definições dos termos também pode ser considerado um dificultador no entendimento e consequente aplicação na prática profissional[16;17].

Para o presente estudo, SAE e PE são distintos. SAE organiza o trabalho profissional quanto ao método, pessoas e instrumentos, tornado possível a implementação do PE[11]. Já PE trata-se de um processo dinâmico voltado para as ações do cuidado na prática profissional, visando identificar problemas de saúde, planejar, implementar ações e avaliar os resultados. Também pode ser definido como uma ferramenta intelectual da prática profissional do enfermeiro, que direciona a lógica clínica e as decisões no que tange a diagnósticos, intervenções, resultados e avaliações. Um modelo de pensamento e atuação que é documentado[14].

Embora seja um processo dinâmico e nem sempre linear, para fins didáticos e permitir melhor compreensão, a Resolução COFEN 358/2009 descreve que o PE é organizado em cinco etapas subsequentes e inter-relacionadas: histórico; diagnóstico; planejamento; implementação e avaliação ou evolução de Enfermagem. Estas etapas são definidas a seguir.

A ***Coleta de Dados de Enfermagem*** ou ***Histórico de Enfermagem*** é a coleta de informações do sujeito, família ou coletividade de forma ordenada, organizada e longitudinal, bem como o uso da observação direta do profissional, utilizando métodos

e técnicas diversas. As informações coletadas nessa fase são significativas para o raciocínio clínico do enfermeiro, visto que possibilitam identificar problemas e potencialidades[11].

Alguns estudiosos dão o nome de "investigação", na qual se insere anamnese e exame físico com o objetivo de identificar problemas e necessidades do paciente e assim verificar seu estado de saúde. Portanto, subdividem essa etapa em cinco passos: coleta dos dados; validação dos dados; agrupamento dos dados; identificação de padrões e comunicação ou registro dos dados"[18]. Outros acrescentam que a coleta de dados tem "propósito" e "direção", além de estar baseada no domínio profissional do enfermeiro. O contexto situacional das relações, a experiência clínica e a capacidade cognitiva do enfermeiro influenciam a qualidade desta fase[14].

A fase seguinte, o ***Diagnóstico de Enfermagem (DE)***, é um processo de desvelamento das informações coletadas na etapa anterior, bem como o agrupamento destas informações, emergindo daí a tomada de decisão sobre as necessidades do paciente e família, fazendo uso de conceitos diagnósticos de enfermagem[11].

Constitui a base para escolha das ações ou intervenções no curso do processo saúde-doença ou ciclo de vida do indivíduo, família ou comunidade com objetivo de alcançar os resultados esperados[18]. Também pode ser definido como julgamento clínico do profissional, baseado nas informações obtidas pela interface com as pessoas, famílias ou comunidade, considerando como estes se portam diante de situações ou eventos e quais significados atribuem a estes. Pode ser dividido em duas fases: *processo* e *produto*. O processo envolve raciocínio, experiência clínica e intuição, bem como necessita de lógica, reflexão e intenção. Quanto ao produto, é a etapa de nomeação dos diagnósticos, ou seja, nomear o fenômeno que requer intervenção ou acompanhamento. Pode-se fazê-lo utilizando um sistema de linguagem padronizada. Existem vários sistemas de linguagem padronizada específicos da enfermagem podendo servir como exemplo e mais divulgados NANDA Internacional, Inc. (NANDA-I), Classificação Internacional das Práticas de Enfermagem (CIPE). Além de outras linguagens como a Classificação Internacional da Atenção Primária (CIAP)[14].

A etapa de ***Planejamento de Enfermagem*** visa a escolha dos resultados que se deseja alcançar e as respectivas ações de enfermagem para que os

resultados ocorram. Isto baseado nas respostas da pessoa, família ou coletividade humana em um dado momento do processo de saúde/doença ou processo de vida, identificadas na etapa de Diagnóstico de Enfermagem (DE)[11].

Alguns autores reforçam a importância de realizar o planejamento de enfermagem após construção do diagnóstico e estabelecer as prescrições baseadas nos resultados esperados. Referem algumas características para que os resultados sejam considerados adequados, tais como serem claros, objetivos, centrados no paciente, estarem relacionados ao diagnóstico, serem alcançáveis, conterem limite de tempo e serem mensuráveis[18]. Essa etapa envolve diferentes atores, incluindo as pessoas sob os cuidados do enfermeiro, a família, a equipe de enfermagem e de saúde, bem como equipamentos envolvidos nesse processo de planejar[14].

A ***Implementação*** é a realização das ações ou intervenções propostas na fase de planejamento[20], devem ser bem escritas a fim de especificar o que a equipe deve fazer e como, além de evidenciar a anotação das ações propostas[27], também pode ou não envolver outros atores para que se coloque o plano em funcionamento, e estar dependentes da prescrição de enfermagem ou de outros profissionais, ser de cuidado direto ou indireto. Vale ressaltar a importância da anotação das intervenções e observações realizadas por qualquer membro da equipe envolvido nesse cuidado, para fomento da avaliação futura[14].

A ***Avaliação*** ou ***Evolução de Enfermagem*** é uma avaliação global da prescrição de enfermagem implementada, considerando as repercussões da assistência na condição do paciente, família ou comunidade. Deve demonstrar o processo dinâmico apresentado pelo paciente. Geralmente ocorre um registro realizado após a reavaliação do estado geral do paciente em 24 horas[19]. Por outro lado, é válido ressaltar que o enfermeiro não deve ficar preso a esse período de 24 horas, considerando períodos mais curtos ou mais longos, sempre que necessário e conforme o seu contexto de atuação.

O PE é privativo do enfermeiro, cabendo a ele a liderança no seu planejamento, execução e avaliação em todos os ambientes onde ocorra o cuidado de enfermagem[11]. Também é responsabilidade e dever dos profissionais da Enfermagem registrar, no prontuário do paciente e em outros documentos próprios, os dados coletados para assegurar reavaliações da assistência prestada, assim como a continuidade do cuidado, o que melhora a qualidade e a segurança deste. Estes dados compreendem um resumo de informações coletadas sobre a pessoa, família ou

comunidade, em determinado momento do processo saúde doença, diagnósticos de enfermagem proposto acerca das repostada obtidas na coleta de dados, ações ou intervenções de enfermagem e os resultados como consequência das ações realizadas[20].

1.4. Processo de Enfermagem como legislação

O PE é parte integrante de uma legislação que confere sustentação legal à profissão de enfermagem.

A partir da década de 1920, a entrevista de enfermagem foi considerada como prógona da consulta de enfermagem, quando, já no fim da década de 60 foi de fato chamada de consulta de enfermagem e compreendida como atividade direcionada prioritariamente ao grupo materno-infantil. A posteriori passou a abranger outros grupos inscritos nos programas de saúde como: programa de controle da tuberculose, da hanseníase e de outras doenças como diabetes e hipertensão arterial[21].

A princípio, o PE ainda denominado CE foi legalizada pela Lei nº 7.498/86 que regulamentou o Exercício da Enfermagem e estabeleceu essa atividade como privativa do enfermeiro[22]. Desde então, tem sofrido mudanças por diversas portarias e resoluções de diferentes instâncias, inclusive do Conselho Federal de Enfermagem.

O Decreto nº 94406/87, em seu artigo 11º, dá legitimidade e determina a consulta de enfermagem como forma de prestação de assistência direta ao cliente e atividade privativa do enfermeiro[23]. A Resolução COFEN-159/93, artigo 11º, que determina como obrigatória a consulta de enfermagem em todas as esferas de atenção à saúde e desenvolvimento da assistência profissional tanto em instituição pública quanto privada e regulamenta as ações do enfermeiro na consulta, prescrição de medicamentos e solicitação de exames[24].

Já na Resolução COFEN 272/2000, passa-se a legislar sobre Sistematização da Assistência de Enfermagem (SAE), além do emprego da expressão “Processo de Enfermagem”[25].

Já a Resolução COFEN 358/2009 fala sobre a SAE e a implementação do PE em todas as instâncias onde há cuidado de enfermagem. Esta considera que a SAE é organizadora do trabalho da enfermagem relativo ao método, aos recursos humanos e instrumentos e que isso torna possível a realização do PE, sendo este último um instrumento metodológico para a orientação do cuidado profissional de enfermagem

e a documentação pertinente. A Resolução COFEN 359/2009 e a Resolução 429/2012 referem-se aos registros de toda a assistência prestada e ao PE[11;20].

1.5. Atenção Primária à Saúde

Para melhor compreensão do cenário estudado, é importante explorar um pouco mais o Sistema Único de Saúde (SUS), em especifico a APS e a inserção da enfermagem nesse modelo técnico- assistencial, embora historicamente a enfermagem se insira na saúde pública antes da existência do SUS.

O acúmulo de discussões, após décadas de lutas da comunidade de técnicos e movimentos populares acerca de uma proposta nova e universal para a saúde, culminou naquilo que se denominou Projeto da Reforma Sanitária. Assim, via Constituição Federal, institui-se um sistema público de saúde chamado SUS[26].

O SUS possui algumas características que o tornam peculiar enquanto sistema de saúde, iniciando pelo fato de colocar a saúde como direito de todos e dever do Estado. Além disso, tem como objetivo: identificar e socializar os condicionantes e determinantes da saúde; formular políticas de saúde que promovam os campos econômicos e social com intuito de dirimir riscos e agravos, bem como construir ações para promoção, proteção, recuperação da saúde, integrando assistência curativa e preventiva. Também tem característica reguladora, fiscalizadora, controladora e executora de ações e políticas relativas à saúde[26].

O SUS também possui Diretrizes e Princípios fundamentais que podem ser divididos didaticamente em técnico-assistenciais e técnico-gerenciais. Os técnico-assistenciais são: universalidade, equidade, integralidade, intersetorialidade, direito à informação, autonomia das pessoas, resolutividade e base epidemiológica[26]. E as Diretrizes e Princípios técnicos gerenciais que dizem respeito à organização administrativa do sistema são: descentralização; regionalização; hierarquização; gestor único em cada esfera de governo; conjugação de recursos das três esferas de governo; organização dos serviços para evitar duplicidade; complementariedade e suplementariedade do privado; financiamento tripartite e participação da comunidade[26]

Quanto à concepção de APS, historicamente, a ideia foi usada pela primeira vez em 1920, em documento do governo Inglês, denominado Relatório Dawson, como forma de se opor ao modelo curativo Norte-americano vigente na época. Esta

concepção influenciou a organização de vários sistemas de saúde ao longo do mundo com duas características básicas: a regionalização, com serviços distribuídos a partir de bases populacionais, e a integralidade, integrando ações curativas e preventivas. Ao longo das décadas foi substrato para discussões da Organização Mundial de Saúde em Alma Ata e Otawa[27].

No Brasil, a APS insere-se no SUS como uma estratégia de organização e reorientação do modelo assistencial, voltada para responder à maior parte das carências de saúde de forma regionalizada, contínua e sistematizada, combinando ações preventivas e curativas, bem como a atenção a indivíduos e comunidades. Atualmente, a APS se configura pela Estratégia de Saúde da Família (ESF), que se aprofunda nos processos de territorialização e responsabilização sanitária das equipes de saúde. Estas equipes se compõem basicamente por médico generalista, enfermeiro, auxiliares de enfermagem e agentes comunitários de saúde, que atendem uma população adstrita[27].

Usa-se aqui o conceito de APS sendo análogo ao conceito de Atenção Básica a Saúde (ABS), embora existam discordâncias na literatura quanto à similaridade entre ambos os conceitos[27]. Assim, a ESF foi constituída de forma que o município passasse paulatinamente a ser o ator principal nas ações de saúde locais, visto que um dos princípios norteadores dos SUS é a ideia de que os problemas locais são melhores entendidos por sujeitos locais, no intuito de fazer com que as ações possam ter maior resolutividade. Tal estratégia foi inspirada por experimentações de municípios e estados brasileiros, como o Programa de Agentes Comunitários de Saúde (PACS), no Estado do Ceará desde 1987, e o Programa Médico de Família, desenvolvido a partir de 1992 em Niterói (RJ). Também influenciados por modelos internacionais, oriundos da Europa e de países como Cuba e Canadá[28].

Em Campinas, a inserção da ESF teve início em 1998 de forma tímida, mas com organização tradicional. A partir de 2001, com uma característica própria e utilizando os recursos já existentes para incorporar novos rearranjos de equipe, criou-se o Programa Saúde da Família – Paidéia para toda a rede municipal de saúde, que tinha como desafio a mudança de modelo de atenção à saúde em uma cidade de grande porte, com um sistema já instalado e funcionando[28;29;30].

Com relação à enfermagem na saúde pública, partiu-se do cuidado de algumas doenças e agravos, como os Programas de Combate à Tuberculose com atividades de dispensação de medicamentos, visitas domiciliares e controle do tratamento de

pacientes[31], para a realização um trabalho voltado para supervisão e treinamentos. O enfermeiro passou a gerenciar o trabalho dos membros da equipe de enfermagem, bem como ser responsável pela avaliação de risco de pacientes dos programas. Assim, houve a expansão e diversificação do trabalho dos enfermeiros para outras áreas como saúde da criança, gestante, imunização e gerenciamento[32].

Especificamente, na saúde pública de Campinas o trabalho dos enfermeiros seguiu o movimento de crescimento da rede básica de saúde, mudanças do modelo técno-assistencial e de políticas públicas voltadas à saúde, havendo um incremento da prática e ações executadas pelos enfermeiros. Estudo realizado em 2008 retrata as atividades desenvolvidas pelas enfermeiras de Campinas, que se constitui numa gama de 1259 ações auto referidas[29].

Nesta era de avanços tecnológicos, mais do que nunca os profissionais precisam mostrar que suas intervenções no cuidado fazem diferença nos resultados, pois quem busca o serviço de saúde necessita sentir-se seguro com as condutas escolhidas[33]. Assim, o PE foi concebido como uma ferramenta importante para dar visibilidade ao cuidado de Enfermagem.

Pensando-se em todas as considerações já expostas, o pensamento crítico se faz cada vez mais necessário ao enfermeiro, devido às rápidas mudanças relativas à atenção à saúde e a crescente complexidade das demandas do sistema de saúde, tanto no que se refere à atividade prática do enfermeiro enquanto ação efetiva sobre a pessoa, família, comunidade quanto à legislação. O pensamento crítico envolve habilidades e atitudes para o raciocínio clínico que interferem nas ações e decisões assistenciais do enfermeiro[34].

Este panorama demanda investigações para entender como favorecer a implantação do PE, visto que ele contribui para o pensamento crítico, aperfeiçoa, responsabiliza e aumenta a satisfação profissional, qualifica a assistência, além das questões legais envolvidas. Desta forma, torna a profissão mais visível, legítima e autônoma, o que possibilita melhorar a qualidade do cuidado, permitindo ao enfermeiro sistematizar suas ações e delegar tarefas à equipe de enfermagem de forma clara e organizada[14;35].

Portanto buscar compreender quais são as percepções que os enfermeiros possuem sobre PE pode oferecer subsídios para novas estratégias de implementação efetiva na atenção primária e, consequentemente, aprimorar a qualidade da assistência, incentivando a autonomia profissional.

A experiência clínica remete a pensar que ainda temos pouca adesão à implantação do PE na atenção primária, o que se expressa por meio da atuação coerente dos enfermeiros quanto às necessidades dos pacientes, mas de forma não sistematizada e sem os registros pertinentes. O que vem acontecendo, apesar de inúmeros avanços e situações que deveriam facilitar a implantação.

Entende-se que tal implementação será mais uma evolução da Enfermagem como profissão, com uma prática organizada e científica, inserida em um modelo assistencial que é voltado para a atenção primária. Não se pode desconsiderar o fato do PE ter legislação em Conselho de classe e função social, bem como o reconhecido valor das informações que são trocadas entre enfermeiro e paciente, relacionadas ao encaminhamento da efetivação do cuidado. Desta forma, o desenvolvimento de um estudo que possibilite compreender as percepções dos enfermeiros da atenção primária quanto ao PE, pode dar luz à pouca adesão a ele na prática clínica e suscitar estratégias futuras que modifiquem este cenário.

2.OBJETIVOS

2.1. Objetivos Gerais

Este estudo tem como objetivo compreender a percepção de enfermeiros a respeito do Processo de Enfermagem na atenção Primária da Secretaria Municipal de Saúde de Campinas.

Descrever os fatores dificultadores e facilitadores na aplicação do PE na APS

3.PERCURSO METODOLÓGICO

Trata-se de um estudo qualitativo e exploratório, que utilizará a perspectiva do referencial teórico do Processo de Trabalho, proposto por Ricardo Bruno Mendes Gonçalves e Maria Cecilia Puntel de Almeida[36,37].

Pelas descrições realizadas acerca deste trabalho, o método qualitativo se mostra adequado e atende aos objetivos desta pesquisa que busca compreender as percepções dos enfermeiros quanto ao PE.

Destaca-se que o estudo de abordagem qualitativa, se caracteriza como reflexivo e interpretativo, pois possibilita considerar múltiplas perspectivas dos participantes, identificando variáveis não mensuráveis, escutando o outro e minimizando as relações de poder. Este método possibilita ainda compreender o contexto em que os participantes do estudo abordam a questão/problema[38].

As matérias primas da pesquisa qualitativa compõem-se por um conjunto de substantivos que possuem sentidos complementares como experiência, vivência, senso comum e ação. Sua análise baseia-se no compreender, interpretar e dialetizar. Também para esta autora o verbo principal da análise qualitativa é o “compreender” e na busca da compreensão é preciso exercitar o entendimento das contradições e conflitos na ação e linguagem pelos efeitos do poder, das relações sociais e dos interesses[39].

3.1. Cenário de Estudo

O estudo se desenvolveu no município de Campinas, interior do estado de São Paulo, Brasil. Munícipio surgido na metade do século XVIII como um bairro rural da vila de Jundiaí, nas margens de uma trilha para as minas dos Goiases. Com o adensamento da população, aumento do comercio e terras férteis, evoluiu de pouso de tropeiros para vila onde foram instalados engenhos de açúcar e plantações de cana. Assim, foi transformada em cidade de Campinas em 1842, período em que os canaviais estavam sendo rapidamente substituídos pelos cafezais, incrementando a riqueza da região, o que rendeu a Campinas a alcunha de Princesa do Oeste[40;41].

Com a crise do café na década de 30 Campinas assumiu uma característica mais industrial e de serviços aliada a novas frentes de agronegócios, somando se a isso novos centros de educação, pesquisa e tecnologia, entre eles a Universidade

Estadual de Campinas (UNICAMP), Instituto de Tecnologia de Alimentos (ITAL) e o Centro de Pesquisa e Desenvolvimento em Telecomunicações (CPQD).

Já na década de 80, ocupava a posição de segundo polo industrial do país em valor de produção, ficando atrás apenas da Região Metropolitana de São Paulo, recebendo a instalação do Instituto Nacional de Tecnologia da Informação, do Laboratório Nacional de Luz Síncontron e da Embrapa Informática Agropecuária e Embrapa Monitoramento por Satélite, fortalecendo uma nova modalidade de desenvolvimento industrial nos campos de informática e telecomunicações[40,41].

Na atualidade desponta como maior polo tecnológico da América Latina, caracteriza-se por combinar serviços, comércio tradicional, produção agrícola, geração de ciência e tecnologia, o que lhe confere destacado lugar político, sendo capital da Região Metropolitana de Campinas (RMC), com 19 municípios e uma população de 2,8 milhões de habitantes. A Região Administrativa de Campinas tem 90 municípios e uma população de 6,2 milhões de habitantes. É o terceiro maior centro industrial do País (após a região metropolitana de São Paulo e do Rio de Janeiro), gerando 3% do PIB brasileiro[40,41].

Com população de 1.173.370 habitantes, Campinas possui grande ocupação urbana e associada a isso enfrenta problemas com proliferação de favelas, pobreza urbana, violência e desemprego. Mesmo assim está ranqueada com um dos maiores índices de desenvolvimento humano (IDH), tanto da região metropolitana como do estado de São Paulo[40].

Campinas também se destaca pela complexidade do sistema de saúde, o que teve início na década de 70. Ainda na mesma década, desenvolvia-se em nível nacional uma grande articulação de movimentos populares e de algumas classes de trabalhadores, trazendo reflexos na estruturação da assistência à saúde da população. Dessa maneira, os Postos de Saúde se originam do movimento popular, principalmente pelas comunidades eclesiais de base e determinados segmentos de técnicos de saúde, parceiros de um movimento encampado pelo Departamento de Medicina Preventiva e Social da Universidade Estadual de Campinas (Unicamp)[30].

Os programas de Medicina Comunitária desenvolvidos pelos Departamentos de Medicina Preventiva da Pontifícia Universidade Católica de Campinas (PUC-Campinas) e da Unicamp, em consonância com as iniciativas das secretarias de saúde dos municípios de Niterói, Montes Claros e Londrina, constituíram-se projetos

de implantação de modelos alternativos de atenção à saúde no movimento sanitário, contribuindo substancialmente para o processo de Reforma Sanitária brasileira. Neste cenário, Campinas usou de recursos próprios para a ampliação da rede de postos de saúde no modelo de medicina comunitária e da participação popular[42].

Possuindo hoje 64 unidades básicas de saúde, Sistema de urgência e emergência constituído de três Pronto Atendimentos (PAs) e Serviço de Atendimento Móvel de Urgência (SAMU), e outras unidades que prestam atendimento secundário e terciários e unidades meio, tanto próprias como contratadas e conveniadas que prestam serviço de diversas especialidades e natureza[40].

Por ser um Centro de Referência regional para o setor saúde, Campinas também absorve a demanda da região, sobrecarregando o seu próprio sistema municipal, tanto na atenção básica, como na assistência secundária e terciária[1].

Toda essa complexidade do sistema de saúde do município levou a distritalização da saúde, que é um processo de descentralização do planejamento e gestão da saúde que se iniciou com atenção básica e posteriormente seguido pelos serviços secundários e conveniados/contratados.

A distritalização se deu em cinco áreas geográficas da cidade com aproximadamente 200.000 habitantes cada, que são Norte, Sul, Sudoeste, Noroeste e Leste (figura 1). Com coordenações independentes em cada um dos distritos de saúde, sendo os coordenadores responsáveis pela organização dos serviços de saúde dos respectivos distritos[1].

Como exposto nos parágrafos anteriores a cidade de Campinas é um município com grande população e extensão geográfica, com sistema de saúde bastante organizado e consolidado e tendo este estudo enfoque qualitativo escolheu-se o Distrito de Saúde Leste para aplicação das entrevistas. A definição deste Distrito para a coleta de dados se deu por conveniência, devido a indicação de profissionais da própria SMS de Campinas, por se tratar de um cenário em que não houve todo o processo de discussão sobre PE como realizado nos outros Distritos de Saúde.

Distrito de Saúde Leste, que se localiza na região correspondente da cidade: possui 10 Unidades Básicas de Saúde com 30 enfermeiros, bem como a Vigilância em Saúde, Centro de Apoio Psicossocial (CAPS) 3, CAPS Álcool e Drogas, CAPS Infantil, Serviço de Atendimento Domiciliar (SAD), Ambulatório de Especialidade, possui uma população de 246.866 habitantes, distribuída em mais de 300 bairros[1].

Figura 1 – Mapa de Campinas segundo Distritos de Saúde

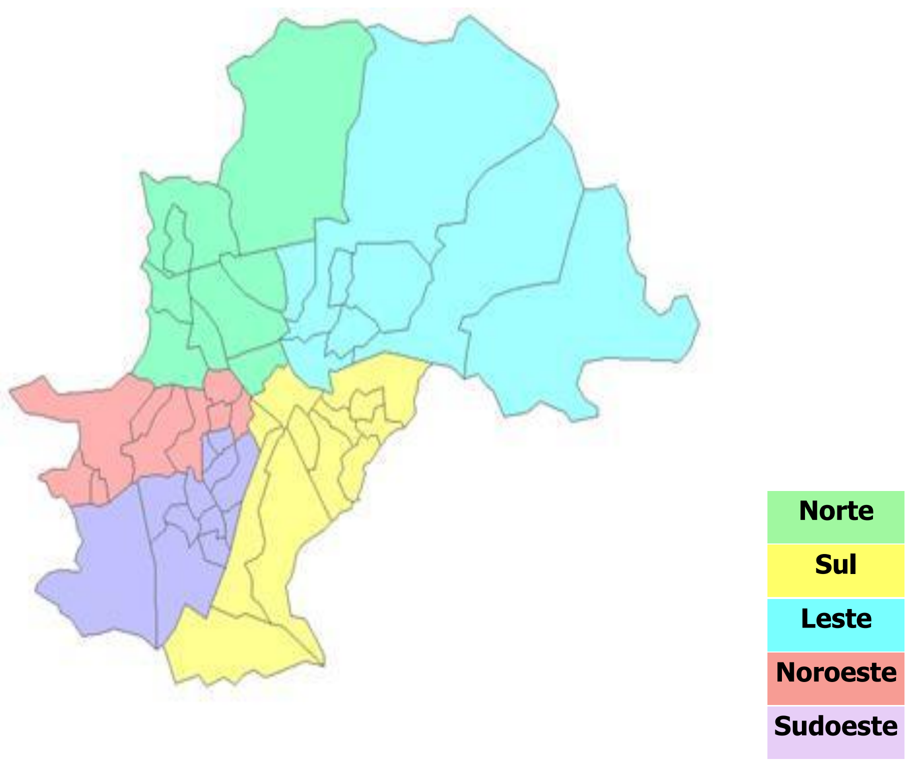

3.2. Sujeitos do Estudo

Como esse estudo envolvia a percepção acerca do processo de enfermagem foram excluídos os enfermeiros em cargo de gestão; enfermeiros que não atuam na atenção primária à saúde; enfermeiros afastados no período de coleta de dados devido a férias ou licenças.

No período de janeiro e fevereiro de 2017 foram realizados os contatos por meio de e-mail, telefones e mensagens via celular, o uso destes métodos de comunicação, principalmente as mensagens via celular foi viabilizado por intermédio de um dos apoiadores do Distrito de Saúde Leste que se responsabilizou por passar as informações de contatos das enfermeiras cadastradas no distrito. Os contatos por e-mail e telefone foram possíveis utilizando os dados institucionais das unidades que constava no site oficial da Prefeitura Municipal de Campinas.

Foram contatadas todas as unidades básicas de saúde via e mail, das quais três responderam, as demais não retornaram mesmo após segundo contato, também foram realizados contatos telefônicos que não se mostraram muito eficazes, pois a maioria das vezes os profissionais não estavam na unidade ou não podiam atender. O melhor resultado se deu por meio de mensagens de celular e contato pessoal após entrevista com algum profissional da mesma unidade.

Importante ressaltar que o início dos contatos e coleta de dados se deu num período de grande instabilidade política na cidade de Campinas: período de início de novo governo, com perspectiva de mudanças de lideranças locais, distritais e nas secretarias, crise financeira com atrasos de pagamentos aos funcionários, falta de insumos e escassez de recursos humanos, principalmente no quadro de enfermeiros, técnicos de enfermagem, técnicos de farmácia, recepcionista e administrativo.

Somando-se à questão da instabilidade política e econômica e demais problemas advindos dessa questão, na segunda quinzena do mês de janeiro de 2017 houveram os primeiros óbitos por febre amarela na região do Sul de Minas Gerais e mortes de macacos nos meses que se seguiram, tanto em Minas Gerais quanto em cidades da região de Campinas. Esta situação epidemiológica desencadeou uma busca pela vacina nas unidades básicas de saúde de Campinas, o que causou sobrecarga nas atividades dos enfermeiros e da equipe de enfermagem que já sofria por falta de recursos humanos. Convém ressaltar que foi no Distrito de Saúde Leste que foram encontrados os primatas não humanos mortos pelo vírus da febre amarela.

Esta sequência de eventos foi parte dos motivos alegados pelas enfermeiras para a recusa da participação. Infere-se que também influenciou negativamente no retorno dos contatos das enfermeiras para com a pesquisadora.

3.3. Instrumento e procedimentos de coleta de dados

O trabalho de campo é um momento bastante importante da pesquisa, revela as preocupações cientificas do pesquisador e não é um espaço transparente ou neutro e todos os seus componentes interferem no resultado daquilo que se pretende conhecer[43].

A coleta de dados foi realizada por meio de entrevista semiestruturada. Tal escolha se deu por avaliar que este método permitiria diálogo mais aprofundado entre participante e pesquisadora.

Além do exposto acima, deve-se ter em mente que a entrevista permite obtenção de dados de duas naturezas, uma mais objetiva como censos, registros e outros dados que poderiam ser adquiridos por outra fonte e outra subjetiva, mostrando ideias, valores, opiniões. Assim, obtêm-se informações mais profundas da realidade, o que só é possível com a ajuda dos atores sociais envolvidos nesse processo[43].

Também pode-se considerar a entrevista em seus diversos aspectos como uma técnica de interação social, interpenetração informativa, capaz de quebrar isolamentos grupais, individuais e sociais, que objetiva permitir a pluralização de vozes, cuja finalidade é o inter-relacionamento humano[45].

A entrevista semiestruturada contem roteiro previamente elaborado, composto por questões abertas que permitem flexibilidade e ampliação dos questionamentos enquanto as informações vão sendo fornecidas pelo entrevistado[45].

As entrevistas ocorreram nos meses de fevereiro e março de 2017, após anuência do Comitê de Ética em Pesquisa (CEP). Previamente à coleta de dados foi realizado teste do instrumento com dois enfermeiros que não participaram da pesquisa, com objetivo de avaliar como seria a experiência da entrevista com os enfermeiros, bem como a pertinência e a clareza das questões, considerando o objetivo do estudo.

Todos os enfermeiros foram entrevistados em suas unidades de trabalho. Eles escolheram data e horário para tal, sendo respeitadas as características de cada unidade e participante. As entrevistas foram realizadas em espaço privativo, em encontro único e gravadas em áudio utilizando aplicativo próprio em celular com 11 minutos de tempo médio de entrevista.

Também foi produzido um diário de campo onde foram registrados dados acerca das impressões e observações realizadas pela pesquisadora, tanto sobre a entrevista em si como sobre os entrevistados, as unidades e fatos históricos, políticos e sociais que poderiam interferir no resultado dessa pesquisa.

A entrevista se iniciou com a seguinte frase: “Fale-me sobre sua experiência com o Processo de enfermagem na atenção primária. ” Que foi seguida, no processo da entrevista por duas questões norteadoras: “Como você percebe o Processo de Enfermagem para a qualificação profissional? ” E “ Como você definiria Processo de Enfermagem? ” Quando ocorria falas vagas ou inespecíficas, seguidas por longo silêncio, foram abordados com um pedido de maior explicitação do pensamento, por

meio de expressões como: "Fale mais sobre isto". Assim, conduziu-se a entrevista de forma a permitir que o entrevistado ficasse à vontade ao longo de seu relato.

O instrumento de coleta de dados apresentou uma parte inicial com dados para caracterização dos sujeitos e uma segunda parte que contempla o roteiro de entrevista (Apêndice 1).

3.4. Considerações éticas

Esse estudo atendeu a Resolução 466/2012 do Conselho Nacional de Saúde (CNS), que regulamenta pesquisas com seres humanos. Assim, a coleta de dados só ocorreu após apreciação e aprovação do projeto pela SMS (anexo A) e pelo CEP da Universidade Estadual de Campinas (UNICAMP), CAAE nº 61606316.0.0000.5404 (Anexo B).

Os enfermeiros foram orientados quanto aos objetivos e procedimentos do estudo, sendo que participaram voluntariamente, leram e assinaram o Termo de Consentimento Livre e Esclarecido (TCLE), em duas vias, das quais uma ficou em poder do participante (Apêndice 2).

3.5. Processamento e Análise dos Dados

As entrevistas foram transcritas integralmente, considerando as falas, as pausas e os detalhes. Após a transcrição, o material foi submetido à "Análise do Conteúdo", modalidade Análise Temática, preconizada por Minayo[39,43].

A Análise Temática é um modo de desvelar os "núcleos de sentido" que fazem parte da comunicação e cuja presença pode significar algo para o objeto que se quer analisar, na pesquisa qualitativa a existência de determinados temas desvela valores de referência e modelos comportamentais presentes no discurso[39,43].

Operacionaliza-se em três momentos ou etapas, sendo estas**:** ***a pré-analise, a exploração do material e o tratamento dos resultados obtidos e a interpretação***[43]**.**

Pré-Analise: Momento de escolha dos documentos que se deseja analisar, com intuito de retomada das hipóteses e objetivos iniciais da pesquisa, reformulando os com base no material coletado e criando indicadores que norteiem a interpretação final[43], assim objetiva a sistematização para que o analista possa conduzir as operações sucessivas de analise[43].

É contemplada por subdivisões ou ações das quais integra-se a ***leitura flutuante***, que consiste na leitura exaustiva do material, promovendo a impregnação do conteúdo pelo pesquisador. Neste momento há uma aproximação dos objetivos e do referencial teórico proposto emergindo temas centrais e outros aspectos que o pesquisador julga pertinente. Depois segue-se **c*onstituição* do *corpus*** que se entende por organização do material que permitam responder algumas normas de validade como exaustividade, representatividade, homogeneidade e pertinência, ***formulação da hipótese e objetivos que*** prevê que as ideias iniciais permitam novas hipóteses a partir de procedimentos exploratórios. Determina-se aqui as unidades de contexto, os recortes, a forma de categorização, a modalidade de codificação e os conceitos teóricos mais gerais que orientam a análise[43].

Exploração do material: nesta fase ocorre a operação de codificação para alcançar o núcleo de compreensão do texto, recortando-se o texto em unidades de registro que podem ser palavras, frases, temas ou um fato e se realiza a classificação e a junção dos dados que possibilita a escolha das categorias teóricas ou empíricas que darão direção aos temas[43].

Tratamento dos resultados obtidos e a interpretação: a partir dos resultados brutos realiza-se inferências e interpretações baseando se no quadro teórico previsto ou que sugere a leitura do material.

O processo de análise de dados em uma pesquisa qualitativa é um processo dinâmico, intuitivo-dedutivo, sendo um movimento entre empírico e teórico e vice-versa, alternando entre o concreto e o abstrato, geral e particular, tratando se de um verdadeiro movimento dialético objetivando a concretude do pensamento[39;43].

4.REFERENCIAL TEÓRICO

O referencial teórico escolhido para oferecer suporte à discussão dos resultados foi Processo de Trabalho, por entender que este referencial possibilita dar luz à questão sobre a prática da enfermagem para além do conceito de profissão, constituída por universalidade, racionalidade, autoridade e competências especificas que possui um estatuto formal e legalizada. Toma a enfermagem e a saúde como trabalho inserido num contexto histórico e social que interage com outras profissões em cenários institucionais e sociais e atendem a uma finalidade ou necessidade[36,37].

Segue uma discussão sobre o referencial proposto de acordo com a gênese teórica de Ricardo Bruno Mendes Gonçalves e Maria Cecilia Puntel de Almeida. Estes autores utilizam a categoria Trabalho para compreensão do processo da saúde. Maria Cecilia Puntel de Almeida faz essa análise mais voltada para a enfermagem, mas ambos seguem uma abordagem marxista da saúde, pautados nas produções de Maria Cecilia Ferro Donnangelo, utilizando seus estudos sobre o trabalho médico, cujo cotidiano das práticas era predominantemente guiado por uma visão positivista, centrado na doença e influenciada pelo modelo de produção capitalista[36,37].

Por tratar-se de um referencial que se utiliza de vários conceitos e busca na historicidade e desenvolvimento social a explicação do processo de trabalho nas práticas de saúde, práticas de enfermagem e suas tecnologias, propõe-se uma divisão didática do assunto que garanta melhor exploração dos temas: Processo de trabalho em Saúde; Processo de Trabalho e a Enfermagem e Organização Tecnológica do Trabalho em Saúde.

4.1.O Processo de Trabalho em Saúde

A categoria trabalho foi introduzida para compreender o processo saúde-doença e as práticas de saúde em nossa sociedade, por entender que a saúde é um trabalho, pois atende a um proposito, um fim ou uma necessidade. Assim sendo, o processo de trabalho é intencional, ou seja, transforma o “antes” em “depois” mediado por um gasto energético. Este “depois” é necessário por se tratar da finalidade desse processo. Essa energia empregada para a transformação do antes em depois caracteriza-se por um conjunto de qualidades ou potencialidades humanas naturais que se ativam para obtenção de transformações, denominada força de trabalho.

Consequentemente, esta força gera energias mecânicas e intelectuais, sendo as mecânicas controladas pelas intelectuais, cuja atividade coordenada de ambas, mediada por instrumentos, com o intuito de obter uma finalidade denomina se “trabalho”[37].

O processo de trabalho finalizado gera um resultado idealizado pelo trabalhador, no entanto, o autor destaca que na disponibilidade de instrumentos, como maquinas e afins, o trabalho humano pode realizar-se sem que o objeto final seja do desejo do trabalhador, o que caracteriza as formas de alienação do trabalho (Marx) que se efetivam nas sociedades capitalistas da contemporaneidade[37].

As necessidades humanas variam conforme o contexto histórico e social nas quais as pessoas se inserem, então afirma-se que neste sentido o homem não trabalha isolado de outros homens, mas como um ser social, portanto as necessidades que, transformadas em finalidades, norteiam todos os processos de trabalho, não são apenas do ser individual, mas também do grupo em que vive[37].

Estas especificidades se dão pelo fato de que, por ser o homem “objeto” do trabalho em saúde, e ao pertencer a determinado momento histórico, é preciso conhece-lo de forma objetiva e subjetiva (emoções, desejos, ódios, necessidades, etc.), que cada um estabelece a seu modo e assim faz de si um sujeito[36].

As necessidades humanas são configuradas de modos diferentes em cada período histórico e assim seus produtos e instrumentos de trabalhos são específicos de cada época.

Ilustrando o que já foi exposto, nas sociedades primitivas os xamãs ou pajés eram responsáveis pelo trabalho em saúde. Eles criam que a doença era uma entidade que possuía o corpo do homem e causava-lhe mal. Este era o objeto de trabalho do xamã ou pajé, e cabia a eles realizar rituais para expulsar tal entidade, portanto, os instrumentos de trabalho aqui utilizados seriam os rituais com objetivo de reintegrar o homem à sociedade[37].

Esta representação da doença para as sociedades ocidentais ocorreu até o fim do século XIII, quando na Grécia essa concepção de rituais é questionada, pois acreditavam que a natureza, incluindo o homem, era o estado de equilíbrio, enquanto a doença, o desequilíbrio. A medicina hipocrática, aqui contextualizada, desenvolveu a classificação do que se chama ainda hoje de clínica, diagnostico e prognostico, sendo clínica o processo de classificação das alterações naturais, diagnostico o

reconhecimento dos tipos de desequilíbrio e prognostico como finalidade do trabalho, para favorecer a natureza a restituir o equilíbrio[37].

Portanto, no momento histórico da Grécia clássica instituiu-se dois tipos de medicina, uma direcionada aos cidadãos livres e outra aos escravos, atendendo as necessidades especificas de cada grupo e, consequentemente, distintas entre si, contribuindo para a reprodução do modo de vida de cada um deles[37].

Na era medieval, cristã e feudal, a saúde e a doença apresentam nova configuração. Assim, a doença passou a ser atribuída ao "pecado" e era o preço a ser pago pelo paraíso. Enquanto as práticas de saúde se transformam na espera da passagem para a vida eterna, com predomínio do trabalho religioso de assistência aos enfermos, sendo essas as necessidades de saúde daquele momento[37].

No século XVI, com a transição do feudalismo para o capitalismo, começa a emergir nova racionalidade e na sociedade capitalista o homem e seu corpo transformam-se na sede da força de trabalho. Por isso, desenrolam-se dois modos de trabalho em saúde: um para o controle de ocorrências de doenças e outro para recuperação da força de trabalho. O primeiro denominado modelo clinico e o segundo modelo epidemiológico, assim organizado em dois polos[37].

4.2.Processo de Trabalho e a Enfermagem

Continuando com a discussão do modelo clinico, individualizante e destinada a recuperar o corpo reduzido a dimensões biológicas e sede da força de trabalho, tendo como instrumento o saber anátomo-fisiológico para dar conta do seu objeto de trabalho, o corpo doente a fim de recuperar o indivíduo[36,37].

No período de transição para o capitalismo, ressalta-se que todo processo de trabalho pode ser desempenhado por um único trabalhador: o médico, possuidor de todas as condições de realizar o diagnóstico e a terapêutica, ou seja, todas as etapas do processo[36;37].

O trabalho médico é separado ora por momentos considerados mais intelectuais ora por momentos considerados mais manuais. Vale citar que até o século XVIII, o médico não realizava seu processo de trabalho em hospitais, mas com as mudanças da pratica foi preciso um espaço adequado para tal e a consequente a ampliação dessa estrutura institucional fez surgir uma necessidade por pessoal que o

organizasse, necessidade de outros trabalhadores, nascendo, assim, um trabalho coletivo[36].

A enfermagem é o agente principal deste trabalho coletivo, mas ainda cabendo ao médico a apropriação do momento mais intelectual do trabalho. Neste modelo clinico, a enfermagem é parte do momento manual do trabalho médico. Aqui nasce a divisão social do trabalho na saúde, posteriormente sendo ampliada também para o trabalho da enfermagem, tendo a enfermeira as funções ditas intelectuais e aos auxiliares e técnicos as funções manuais deste trabalho[36]. Neste contexto, a enfermagem no modelo clinico passa a ter a atividade de cuidar, sendo o corpo o objeto dessa ação, e também a administrar como resultado do processo histórico e social executado pela enfermeira[36].

O local histórico, geográfico e social desse momento de institucionalização da enfermagem reside na Inglaterra do século XVIII, em que a industrialização e a urbanização produziram uma necessidade de disciplina nos espaços institucionais, no caso, o hospital. Estes mecanismos disciplinares foram realizados utilizando-se treinamentos de pessoal para prestar assistência de enfermagem, permitindo disciplinar as condutas e minorar os efeitos negativos do hospital[36]. A tarefa de treinamento foi realizada por Florence Nightingale. Ainda que a ação de educação para o trabalho se desse no modelo clinico, sua atuação era humanística, avançada para a época e tinha uma visão mais integral do ser humano, inserido em uma família e meio ambiente[48].

Neste momento da sociedade capitalista o modelo epidemiológico também se desenvolve voltado para as necessidades vigentes, tendo a doença uma concepção de fenômeno coletivo. Esse desenvolvimento se dá principalmente na Europa Oriental entre os séculos XVII e XIX, período de grandes epidemias, com muitas mortes que foram associadas às características dos pacientes, ao tempo, e ao ambiente em que viviam. Nasce assim a medicina social que tem como objetivo o controle das doenças utilizando se de instrumentos para este controle como saneamento, a higiene social, a polícia médica, a quarentena e as estatísticas de mortalidade. Outros instrumentos eram utilizados para controlar o surgimento de doenças na população, dentre eles a própria clinica[36].

Neste contexto a enfermagem deixa de participar exclusivamente do trabalho médico, inserindo-se no trabalho em saúde e mais especificamente em saúde coletiva.

O que contribui para a produção dos serviços de saúde coletivos cujos saberes e práticas objetivam as carências sociais de saúde da população[48].

Assim definiu-se, no modelo epidemiológico, que os corpos sociais são objeto da pratica médica e passaram a analisar o processo saúde-doença em relação a estrutura social, econômica e ideológica[37;48].

4.3.Organização Tecnológica do Trabalho em Saúde

O conceito de organização tecnológica do trabalho em saúde foi construído também por Mendes Gonçalves[49], referindo se as conexões estabelecidas no processo de trabalho entre a ação proposta e a finalidade, via utilização de instrumentos, dos quais o saber ou conhecimento que conduz o processo de trabalho é um desses instrumentos[49].

O autor usa como ponto de partida para essa discussão uma descrição do conceito contemporâneo acerca do termo tecnologia, sendo "tecnologia", o conjunto de instrumentos materiais, conjunto de coisas e objetos com uma função técnica nos processos de produção. Contudo, Mendes[49] interpreta o, inserindo o "saber" e suas vertentes materiais e não materiais na produção dos serviços de saúde[49].

Portanto, o autor conceitua tecnologia como uma soma de saberes e instrumentos que exprime nos processos de produção de serviços, as inter-relações sociais, cujas praticas são articuladas por seus sujeitos em uma completude social e enfatiza o "saber" descrevendo este como o diálogo entre os resultados do processo de conhecimento e as imposições de outros elementos que surgem para a pratica de sua conexão social. Embora a discussão seja sobre tecnologia e organização tecnológica do trabalho em saúde, o interesse das investigações do autor se volta de forma relevante sobre "saber" enquanto conhecimento[49].

O tema referente ao "saber" também foi objeto de investigação no campo da enfermagem. No livro "O saber de enfermagem e sua dimensão prática", os autores sugerem o estudo da prática e do saber de enfermagem em uma ótica histórico-social, selecionando as técnicas de enfermagem, princípios científicos e as teorias de enfermagem como saberes por expressarem quase todo o arcabouço de conhecimento ou saber de enfermagem[50]. Pensando que segundo o que foi exposto nesse tópico acredita-se que a enfermagem utiliza em seu trabalho tanto instrumentos materiais quanto intelectuais para a realização da sua pratica[50].

Nessa lógica, Merhy aprofunda a concepção sobre tecnologia incluindo outros saberes que são os utilizados na produção dos “produtos singulares” nos serviços de saúde, bem como os saberes que atuam na organização das ações humanas e nas relações humanas nos processos de produção. O termo “saberes” é utilizado como sinônimo de conhecimento, arcabouço teórico ou tecnologia e não foi substituído por ser desta forma que o autor se expressa para descrever todos os termos sinônimos. Estas tecnologias são classificadas como duras, leve-duras e leves, que se caracterizam da seguinte forma[51]:

- **Tecnologias duras:** seriam os equipamentos, as maquinas, resultado de outro momento de produção e conformam em si outros saberes já sedimentados e materializados;
- **Tecnologias leves-duras:** podem referir-se aos saberes que dão direção e sustentação científica ao trabalho como as normas, os protocolos, conhecimentos produzidos em determinadas áreas do saber como a clínica, a epidemiologia, entre outros;
- **Tecnologias leves:** estas são produzidas nas relações de cunho subjetivo que possibilitam produzir acolhimento, vinculo, responsabilização e autonomia dos sujeitos.

Merhy afirma a necessidade de mudanças no processo de trabalho usando como eixo a concretização da tecnologia leve e suas possibilidades de inter-relação com as outras tecnologias[51].

5. RESULTADOS E DISCUSSÃO

5.1. Caracterização dos participantes

Todos participantes prestam assistência em UBS da SMS da Cidade de Campinas. Suas características são apresentadas na Tabela 1.

Tabela 1 – Distribuição das características dos enfermeiros entrevistados. Campinas-SP, 2017

Característica	N	%
**Idade (anos)**		
$\leq$ 30	1	8,3
31 a 40	5	41,7
41 a 50	3	25,0
51 a 60	3	25,0
> 60	-	-
**Sexo**		
Feminino	9	75
Masculino	3	25
**Curso de Pós-Graduação**		
Sim	9	75
Não	3	25
**Tipos de cursos***		
Saúde do trabalhador	2	16,6
Saúde da família	6	50,0
Saúde pública	2	16,6
Gestão publica	2	16,6
Gestão em saúde	2	16,6
Adm. Hospitalar	1	08,3
Urgência e emergência	1	08,3
Cardiologia e hemodinâmica	1	08,3
Formação Pedagógica	1	08,3
Acupuntura	1	08,3
Mestrado (cardiologia)	1	08,3
**Tempo de formação (anos)**		
$\leq$ 5	-	-
6 a 10	3	25
11 a 15	4	33,3
16 a 20	1	8,4
> 20	4	33,3
**Tempo de atuação na PMC** (anos)**		

≤5	1	8,3
6 a 10	5	41,7
11 a 15	-	-
16 a 20	3	25
> 20	3	25
**Tempo de atuação na UBS*** em que atua (anos)**		
≤5	8	66,7
6 a 10	2	16,7
11 a 15	-	-
16 a 20	1	8,3
> 20	1	8,3
**Total**	**12**	**100**

*Alguns participantes cursaram mais de uma especialização

** Prefeitura Municipal de Campinas

*** Unidade Básica de Saúde

Os dados deste estudo demonstram que, trata-se de um grupo jovem em termos de idade para o trabalho, isto segundo a discussão anterior, realizada por Machado[52]. Não houve participantes com 60 anos ou mais. Os dados do Brasil e de Estado de São Paulo apresentam mais que o triplo do percentual de enfermeiros com idade inferior a 30 anos da presente amostra: sendo 25,3% no perfil brasileiro e 23,3% no estadual[53].

As demais distribuições seguem com características que se aproximam aos perfis do presente estudo, considerando os dois citados[52;53]. Segundo Machado 41,7% dos entrevistados estão na fase da formação profissional, que se define por busca de qualificação para os serviços, associada à perspectiva de inserção no mercado de trabalho buscando funções de maior complexidade e destreza cognitiva. Na fase de maturidade profissional estão 39,5% dos enfermeiros, sendo profissionais em uso pleno de suas capacidades, preparados e qualificados para o trabalho que desenvolvem, apenas 12% apresentam-se em processo de desaceleração da vida profissional que tem como característica certa acomodação já se preocupando com a garantia de suas aposentadorias[52].

No que diz respeito a distribuição segundo sexo (Tabela 1), apresenta-se aqui uma porcentagem de profissionais do sexo masculino de 25%, aproximadamente duas vezes maior que no perfil brasileiro e paulista, que são respectivamente 14,4% e 12,8%, apesar da enfermagem ser histórica, cultural e predominantemente feminina,

evidencia-se um crescente aumento da masculinização como tendência nessa categoria[52,53].

No que tange a questão de realização de pós-graduação, apresentam se características bastante aproximadas entre os três perfis: a maioria dos enfermeiros entrevistados em Campinas (75%) possui uma ou mais especializações, seguindo a tendência nacional e estadual[52].

Na tabela 1, nota-se que está em consonância com a fase de maturidade profissional que, para Machado, significa que os enfermeiros se encontram preparados e qualificados para o trabalho. Já como especialistas, possuem mais certeza de que área se firmar enquanto profissional[52].

Os cursos de pós-graduação referidos pelos sujeitos estão relacionados em maior ou menor grau com a atenção primaria e as ações desenvolvidas pelos enfermeiros nas UBS, que embora atendam todas as fases de vida e patologias apresentam núcleo de conhecimentos específicos.

Outro dado importante é que seis dos doze enfermeiros realizaram pós-graduação em Saúde da Família, ou seja, 50% dos entrevistados. Sendo que 16,7% cursaram pós em Saúde Pública, verifica-se que 66,7% dos sujeitos entrevistados se especializaram para o trabalho que realizam na atenção primaria, estando capacitados para tal. Os estudos brasileiro e paulista não investigaram os tipos de cursos realizados pelos enfermeiros[52].

Quanto ao tempo de conclusão do curso de graduação em enfermagem, os dados mostram que não existem sujeitos nessa amostra que possuem 5 anos ou menos de formados, estando 75% formados há mais de 10 anos. Dado interessante se for levado em consideração que os enfermeiros entrevistados, em sua maioria (75%), estão em idade inferior ou igual a 50 anos, corroborando com estudo que descreve quanto os profissionais de enfermagem são jovens[52].

Em relação aos dados paulista, há uma discrepância quanto ao tempo de formação dos profissionais. Não sendo observado no presente estudo indivíduos com 5 anos ou menos de formação, contra 29,6% no estudo paulista[53].

No que se refere ao tempo de trabalho na prefeitura de Campinas, um dos entrevistados tem menos de 5 anos (8,3%), 41,7% dos profissionais atuando na prefeitura entre 6 e 10 anos e 50% com tempo de 16 anos na referida SMS.

Os dados referentes ao tempo de trabalho na UBS atual mostram uma lacuna no intervalo de 11 a 15 anos: não há enfermeiros entrevistados que trabalhem na UBS

por esse período, sendo que a maioria destes (83,4%) trabalham na unidade atual nos últimos 10 anos. Apenas 16,6%, estão acima de 16 anos no mesmo posto de trabalho.

Os dados deste perfil demonstram que os participantes desse estudo possuem experiência na atenção primaria à saúde, capacitaram-se para prestar assistência adequada e desenvolver melhor sua prática.

5.2. Os relatos dos enfermeiros e as categorias temáticas

A partir do material obtido pelos depoimentos dos enfermeiros da Atenção Primária à Saúde, surgiram três categorias temáticas:

- Situações extrínsecas que interferem na realização do PE;
- Situações intrínsecas que interferem na realização do PE;
- O saber do enfermeiro.

5.2.1 Situações extrínsecas que interferem na realização do PE.

Para Puntel[36] a enfermagem é um trabalho social, pois se dá nas relações com outras profissões e em espaços institucionais e sociais diversos, assim a enfermagem não atua sozinha e nem desconectada do todo. Pode-se tomar esse todo como o Mundo, o Brasil, a Prefeitura de Campinas e as respectivas unidades de trabalho, portanto, os acontecimentos de cada "todo" interferem no "um" que é o trabalho da enfermagem e em como sua prática é desenvolvida nos espaços de trabalho. Isto fica claro nas seguintes falas:

> *"[...] porque é.... primeiro porque a gente está vivendo ãh...uma situação difícil no país: essa crise econômica. Então, tudo isso tem um agravante, um impacto direto para a atenção básica como porta de entrada do sistema". (E*5)

> *"[...] mas acho que a gente acaba também ficando desestimulada porque a gente quer fazer algumas coisas e a Prefeitura bloqueia a gente naquilo. " (E4)*

As interferências de ordem nacional e da administração pública municipal são expostas aqui como tendo um papel negativo no trabalho do enfermeiro. A política econômica se interpõe às políticas públicas e de saúde. Desta forma, mesmo que as diretrizes dos modelos assistenciais preconizadas pelas políticas de saúde sejam de inclusão e acesso, a falta de investimento e financiamento adequado faz com que o profissional de saúde seja o responsável pela garantia dessas diretrizes nos serviços de saúde, em especial o profissional de enfermagem. Esta responsabilidade, associada a condições de trabalho inadequadas causa um grande descontentamento e interfere no trabalho diário do enfermeiro.

É preciso reconhecer que o setor saúde vai além da produção desta, em que se entrelaçam agentes, ações, funções, arcabouços legais, gestão, organizações e tecnologias, recursos financeiros e suas modalidades de aplicação[54]. É neste emaranhado que está o enfermeiro, seu trabalho e suas práticas diárias, tendo que dar conta da demanda, das cobranças inerentes a profissão dentro da unidade de saúde.

Outro aspecto cujos enfermeiros julgam causar interferência negativa na realização do PE é a estrutura física local e os vários desdobramentos dessa questão:

> *"Outra coisa que não ajuda, mas eu estou me policiando mais, é a falta de estrutura, então... Para fazer acolhimento eu nem sempre estou numa sala ali...e organizada para fazer aquilo. Às vezes, eu vou olhar uma paciente aqui e outra ali... e aí deixo o relatório para fazer depois, para atender o próximo. Aí quando eu vejo, se perdeu...aí depois... O que eu acho que dificulta é a estrutura e o tempo, acho que talvez, porque não está instituída na nossa cultura. A gente não gosta de deixar muito o paciente esperar e acumular ficha e é isso. "* (E8)

> *"Então, assim, eu não consegui abrir agenda de Papanicolau. Não tenho espaço físico e de demanda de trabalho. [...] A gente não faz porque a gente tem quatro ginecologistas e não temos sala porque a Universidade [cita o nome de uma Universidade] atende todo o período aqui. Se eles não estão, os ginecologistas estão, então a gente não tem sala. " [...] eles vão reformar.... Mas a gente não foi chamada para discutir como que vai ser feita essa reforma: se vai ser feito só uma maquiada ou se, de repente, vai se construir outra sala, se vai melhorar espaço físico".* (E11)

Embora exista uma mudança no modelo assistencial com a proposta do atendimento multiprofissional aos usuários, consultórios ainda são espaços de privilégios, onde o profissional médico continua dominando. Os espaços de gestão, tanto da unidade quanto em outras esferas, não conseguiram incorporar que outros atores possuem saber próprio e autônomo estabelecido e podem atuar também individualmente com o usuário.

Conquanto o SUS esteja vigente em nosso país desde 1990 a sua consolidação vem dependendo do cumprimento efetivo das leis e do interesse concreto dos seus gestores em todas as esferas de governo e, principalmente, no nível local da atenção e é nesse espaço local que fica evidente como se insere o trabalho do enfermeiro e quão importante para a instituição ele é. Também existe uma parcela da problemática levantada quanto a falta de estrutura física que diz respeito, não só aos interesses das esferas governamentais, mas como o profissional enfermeiro percebe seu trabalho e atuação dentro da APS que interfere na divisão do espaço de atuação dentro da unidade de saúde. Esta observação pode ser explicada pelo fato de que ao médico cabe as ações intelectuais na relação com o usuário e a enfermagem cabe a ação mais manual, não sendo necessário um espaço especifico para tal[36].

Colaboram com essa discussão duas pesquisas realizadas após implementação do PSF/Paideia em Campinas afirmam uma tendência no modo de trabalhar dos enfermeiros, passando de gerencial para assistencial, porém ainda ocorre de forma auxiliar ao trabalho do médico[55;56].

Outro aspecto importante é que os enfermeiros se moldam ao contexto, adequando-se ao modelo assistencial[55;56]. Ou seja, o enfermeiro se insere no modelo assistencial, nesse caso a estratégia Saúde da Família, mas não faz deste um espaço de construção da sua autonomia enquanto trabalhador da saúde e da APS.

Outro estudo aponta que a enfermagem trabalha com práticas assistenciais antigas e novas no mesmo ambiente, o que dificulta a mudança do paradigma biomédico e cartesiano, mantendo a enfermagem com olhar segmentado e atrelado ao trabalho médico[56,57].

Outros aspectos abordados pelos entrevistados, que interferem na realização do PE, dizem respeito ao excesso de trabalho e à multiplicidade de ações do enfermeiro, associados à falta de recursos humanos da equipe de enfermagem:

“ [...]algumas coisas estamos em processo de organização, mas sempre tem muito a fazer... porque eu acho que o centro de saúde lida com uma gama de variáveis muito grandes. A tensão é muito grande e são muitas coisas.” (E7)

“[...] O que eu observo é a multitarefa ou multi-funções que o enfermeiro exerce aqui. O enfermeiro se torna líder de uma equipe que não só de uma equipe de enfermagem. E a gente é acessado toda hora, independente do que a gente esteja fazendo, né? Então, a gente é acessada para urgência, a gente é acessada para questões administrativas, a gente é acessada para questões de vigilância que não competem àquele ambiente, digo, àquele momento na verdade. Então, eu entendo que a primeira coisa são as multitarefas que nos deixam, dentro da consulta sendo interrompida a todo o momento[...]” (E12)

“[....] Pelo menos para os médicos eu acho mais fácil porque eles têm aquela quantidade de paciente agendado e aquela quantidade de acolhimento por dia....eles têm um limite. E a enfermagem trabalha com a porta totalmente aberta, você tem que dar conta dessa demanda que vem bater a nossa porta e daí é.... está muito estressante do jeito que a gente está trabalhando desse jeito. ” (E5)

“Bom, o nosso processo de trabalho é.... eu acho assim ... primeiro faltam profissionais, né? O número de RH é insuficiente. Aqui mesmo nessa unidade temos quatro técnicos de enfermagem no momento. [...] aplicar SAE, por exemplo, nós não conseguimos... aqui para nós... a nossa região é mais calma. Mas mesmo assim é pesado...”[E3]

No que tange à falta de recursos humanos, é possível afirmar que está intimamente ligado à situação política e econômica do momento, bem como à falta de investimento pelo qual passa o setor nos últimos anos: fruto de disputas ideológicas. Merhy diz que se faz necessário reconhecer que o SUS se dá num campo de práxis sociais, sob interferências de tensões que o coloca sob disputas sociais e históricas[58].

Corroborando a isso, uma problemática evidente no diz respeito ao financiamento para o setor saúde, que não é suficiente para atender todas as carências deste, o que compromete a resolutividade do SUS, tornado esse assunto um grande desafio[59], tanto nas gestões macro como União, Estados e Municípios, quanto na gestão das unidades de saúde e em especifico no nosso objeto de estudo

que são as práticas de enfermagem, que dependem de recursos para poderem ser realizadas a contento.

Com relação à quantidade de tarefas e ações realizadas pelos enfermeiros pode se dizer que na realidade de Campinas existem algumas características que contribuem para o excesso de trabalho ou “multitarefas” como denominado acima.

A primeira delas refere-se ao fato de que, embora a SMS tenha adotado a Saúde da Família como estratégia para APS, algumas unidades funcionam em horário estendido, das 07 às 18 horas ou 19 horas, algumas até as 22 horas, fazendo com que o enfermeiro seja responsável por mais de uma equipe em seu turno de trabalho. Podendo ser o único enfermeiro na unidade no horário, respondendo pelas demandas de toda unidade naquele momento, o que possibilita questionar se a qualidade da assistência na APS não está se perdendo, ao não se adequar o número de enfermeiros para atender essa realidade.

Outra questão refere se a complexidade da assistência prestada na APS do município e das ações oferecidas nas UBSs, o enfermeiro é o responsável, ordenador e disseminador do conhecimento e saber dessas ações para toda a equipe.

Em consonância ao que foi exposto quanto as inúmeras ações desempenhadas pelo enfermeiro e suas muitas responsabilidades, este também é responsável pelo trabalho do Agente Comunitário em Saúde (ACS) como preconizado no Regimento Interno de Enfermagem de Campinas[1], e, embora não de direito, mas como um fato abordado pelos enfermeiros em outros relatos e reuniões responsabiliza se pelo trabalho do pessoal da limpeza/zeladoria. Entende-se aqui que o enfermeiro é responsável pela organização do espaço institucional, sendo ele hospital ou outro espaço onde as ações de saúde ocorram[36].

Para ilustrar a gama de ações desenvolvidas pelo enfermeiro, em 2008 Marques realizou um estudo que retratou as atividades realizadas pelas enfermeiras deste município, após implantação do PSF-Paideia: 1259 ações foram citadas pelos enfermeiros. Foram agrupadas em três conjuntos de atividade como seguem[29]: “Atividades assistenciais”, “atividades gerenciais” e “outras atividades”.

Nas **atividades assistenciais** foram citadas**:** ação educativa; atendimento individual, execução de procedimentos/técnicas de enfermagem; recepção de usuários e controle de fluxo interno; encaminhamentos e entrega de medicamentos; vigilância em saúde e imunizações; visitas domiciliares. Nas **atividades gerenciais** teve-se: coordenação, organização, treinamento, controle e supervisão do trabalho da

enfermagem; coordenação de áreas, programas e equipes que não a de enfermagem; gerencia conjunta da unidade, atividade gerencial; planejamento, organização e avaliação da unidade. Quanto a **outras atividades:** participação em Conselho Local de Saúde e Colegiado Gestor; atividades não relacionadas a gerencia e a assistência[29].

Outro estudo[33], realizado em 2011 corrobora com os dados acima, identificando atividades semelhantes realizadas por enfermeiras de ESF em cidades do Rio Grande do Sul.

Em outra pesquisa realizada há mais de 10 anos[60], os autores já observavam a sobrecarga de trabalho, tanto pela centralização de atendimentos para a enfermeira, quanto pela falta de recursos humanos na unidade, o que acarreta em insatisfação e desmotivação com relação ao trabalho.

Assim o assunto relativo ao excesso de trabalho e multiplicidade de ações do enfermeiro tem sido abordado por vários estudos em épocas distintas continuando atual e mostrando que essa multiplicidade de ações dentro dos aspectos gerenciais e assistenciais interferem no trabalho do enfermeiro de modo negativo e consequentemente na aplicação do PE.

Dentre a questão levantada que versam sobre o excesso de trabalho uma ação em especial foi relatada nas entrevistas, esta trata se do acolhimento dos usuários como pode se observar a seguir:

> *“[...], mas assim as anotações, nós fazemos acolhimento, né? Então...no acolhimento os dados básicos..., mas está bom, melhorou bastante também...éramos também devagar, mas temos anotado mais coisas.”. (E3)*

> *“[...]A questão do acolhimento... de ser obrigada a passar...ser obrigada a fazer...eles fazem de uma forma para a gente que a gente não tem perna para aquilo...A demanda engole. ” (E4)*

> *“Então, você acaba, nos atendimentos de acolhimento mesmo que é o maior volume de atendimento, você acaba fazendo queixa conduta. Não dá para fazer o processo mesmo, fica muito focal, não dá para...e nas consultas de enfermagem que a gente tem agenda uma única vez por semana é um número menor que daí é quando é possível fazer esse registro um pouco melhor, mais qualificado. ” (E9)*

O acolhimento foi pensado como uma postura dos profissionais para o atendimento aos usuários de forma que se construísse vínculo e permitisse olhar o outro, no caso o usuário, como um ser humano completo, como um encontro, um modo de se relacionar. À princípio pensa-se que o acolhimento tem um lugar indispensável nas relações de diálogo que existem no serviço de saúde, e deveria ocupar todos os espaços, tendo o papel de condutor e conector dos diferentes espaços de diálogo. A ideia central é que todo trabalhador deveria acolher e desencadear um processo de resolução das necessidades expressas pelo usuário e dessa forma aumentar o acesso aos serviços de saúde[61]. E se pensado como tal se pareceria mais a um ritual, porém da forma como foi instituída na maioria das unidades de Campinas, o espaço ou postura de acolhimento mais se parece a uma "esteira de produção, para atender a demanda espontânea que chega as unidades" [54].

Mas, na inserção do acolhimento no processo de trabalho das unidades de saúde da APS em Campinas observa-se que esta atividade ficou a cargo da equipe de enfermagem com supervisão do enfermeiro e em algumas unidades diretamente com o enfermeiro. Transformando-se se numa prática normativa de triagem da demanda espontânea e organização do fluxo dos usuários dentro da unidade.

De certo modo remete aos primórdios da profissão, quando a enfermeira possuía a função principal de organizadora do espaço institucional[36].

Apesar disso observa se nas falas uma intenção ou avaliação da necessidade de aplicar o processo mesmo que isso não ocorra como é preconizado.

A organização do processo de trabalho ou como as coisas são organizados nas unidades e no serviço de enfermagem também foi relatado de inúmeras formas.

> *"Então...não temos em todos os lugares [o processo de enfermagem] por uma falta de...por uma dificuldade até questão do que a gente tem que fazer. Temos que abraçar muita coisa...de discordância de algumas coisas que a prefeitura acha que a gente tem que fazer e que a gente acha que não tem que fazer e um pouco de…deixa para lá que a gente tem deixado também. Acho que é isso.". (E4)*

"Quando eu cheguei aqui em, no CSXX a gente não fazia CO [Citologia Oncótica]. A gente fazia só o atendimento básico, tal...aí a gente foi buscando...a gente...hoje a gente atende algumas pacientes fazendo preventivo, fazendo Pré-Natal. O Pré-Natal sempre inicia com a gente, depois passa em consulta com o médico e vai alternado as consultas, entre o ginecologista e o enfermeiro. Na consulta a gente...na primeira consulta a gente faz o cadastro do SIS Pré-Natal, a gente solicita os exames, orienta a paciente durante todo o Pré-Natal, tudo que vai acontecer com a paciente, a gente, faz a receita, dá repelente e mais orientações..." (E6)

"Mas a gente faz consulta de gestante intercalado com o ginecologista e a gente faz também de coleta C.O. e aí depois, posteriormente no resultado se der alguma coisa a paciente passa no ginecologista. Essa é a organização que a gente tem... uma coisa que eu acho que a gente precisa dar uma organizada boa é em relação à recepção, né? É....a gente não tem uma pessoa...um administrativo, e na verdade é a enfermagem...ela está em todos os setores, né? E na recepção também a gente tem uma certa contribuição, então eu imagino que de repente a gente possa estar ajudando nessa organização lá". (E7)

"[...]um dos motivos é indisponibilidade de agenda, né? Então assim, eu tenho os meus dias programados de agenda. O agendamento é mal feito na minha opinião...é agendado coisas que não necessariamente teriam que vir para o enfermeiro. " (E12)

A organização do processo de trabalho dentro das unidades da APS pode ser um facilitador ou dificultador da implantação do PE. Os enfermeiros relatam que o PE não é feito em todos os atendimentos ou espaços em que o enfermeiro atua. Depreende-se dos depoimentos e da literatura que a falta de recursos humanos na enfermagem, e em outras áreas de interface, comprometem a organização do serviço e consequentemente o trabalho do enfermeiro na unidade.

Na fala de E4 observa-se um conflito entre o que ela acredita que é o trabalho do enfermeiro e são as diretrizes da Prefeitura causando impacto na aplicação do PE em todas as atividades. Outro ponto abordado foi a organização das agendas para o atendimento do enfermeiro, e bem como o papel deste na unidade de APS.

Em seguida um aspecto que chama bastante a atenção são as falas relacionadas à legislação, sendo que estas descreveram apenas as visitas do COREN às unidades:

> *"Estamos no começo agora. Devido a avaliação...a vistoria do COREN, elaboramos todos os processos...é escala das salas...procedimentos e tudo mais. " (*E2)

> *"Bom agora até melhorou um pouco..., mas assim...antes assim não se escrevia quase nada...as anotações eram bem falhas.... Então nós começamos a mostrar né?...o COREN inclusive veio aqui e tal...viu né a necessidade de se escrever, de se identificar né? De carimbar e assinar o que o profissional está fazendo, porque muitas vezes a pessoa vinha ver PA [Pressão Arterial], um exemplo básico...10 vezes no ano e não tinha nenhuma vez anotado. Então isso aí é um documento né? Então está bem melhor as anotações..." (E3)*

> *"Depois que o COREN veio aqui, eu tenho colocado a parte de diagnóstico de enfermagem. Acho que ainda não está a contento, precisa melhorar muito, mas ainda tenho colocado, mas meta, essas coisas eu ainda não coloco, não. " (E8)*

> *"Então é uma coisa que de repente o COREN quer, mas nem os alunos novos que estão se formando dominam isso[...]"* (E11)

Chamou a atenção a inexistência de falas que apontam o PE como parte da legislação. Muito menos foi identificado como ciência que consagra a enfermagem enquanto profissão instituída. O PE norteia o raciocínio clínico do enfermeiro em sua tomada de decisões, bem como favorece o registro do cuidado realizado e de seus resultados: o que parece não ser reconhecido pelos enfermeiros com regras e modos de fazer e agir legalmente. Observa-se que este é um resultado muito sintomático no que tange ao fato de como a profissão de enfermagem se observa e se entende. Faz uma subutilização do registro do PE e de outros registros pertinentes a legislação profissional diminuindo a visibilidade à assistência de enfermagem.

Segundo E11, a falta de domínio sobre o PE ocorre com alunos de graduação que o tem como formação específica. A formação dos futuros enfermeiros para aplicação do PE também é uma preocupação.

As falas se concentram em apontar o COREN como órgão fiscalizador externo, sem conexão com a pratica clínica do dia a dia. Os enfermeiros incorporam algumas atividades como uma obrigação que realizam apenas por receio das notificações, como se esse conjunto de leis existentes não fossem percebidos pelos enfermeiros da APS como representante de suas práticas cotidianas.

A análise das falas trouxe à tona a percepção dos enfermeiros quanto às interferências externas que limitam a realização do PE em sua prática clínica.

Ressalta-se que olhar para a enfermagem com a lente do trabalho mostra que os enfermeiros entrevistados estão num momento de sua inserção na APS que necessita se fortalecer como trabalho e profissão. Mesmo, a enfermagem tendo uma história de longa data na saúde pública e inserção importante na APS, não houve incorporação de processos de autonomia profissional da forma que poderia e necessitaria ser.

Nesse sentido, tais enfermeiros percebem o PE como importante, mas não prioritário nesse momento, sendo que outras necessidades o antecedem. Os entrevistados percebem que se faz necessário o fortalecimento do SUS, com melhoria no financiamento que possibilite investimentos para contratação de mais profissionais e melhorias nas condições e processos de trabalho. A falta dos aspectos citados impacta negativamente na inserção do PE na pratica diária do trabalho do enfermeiro.

Faz -se aqui uma reflexão sobre a potência do PE como instrumento para melhora da organização tecnológica do trabalho do enfermeiro e da equipe de enfermagem pois permite, quando utilizado corretamente, conexões entre a ação proposta e a finalidade. Podendo ser encarado como instrumento de ordem intelectual ou relativo ao conhecimento. O fato de não ser relatado como parte do trabalho do enfermeiro ou parte legal da profissão faz com que essa tecnologia ou saber não seja apropriada pelos enfermeiros para fortalecimento da profissão e qualidade da assistência.

5.2.2 Situações intrínsecas que interferem na realização do PE.

Nesta categoria foi possível realizar uma conexão com o tópico referente a legislação, abordado anteriormente e iniciar essa categoria de analise relatando as falas referentes a construção do PE e suas características e estrutura legais.

“Eu acho que ele [o PE]... na atenção básica ele é meio...como vou dizer...dentro do hospital é mais fácil da gente aplicar porque você está vendo o paciente todo o dia... Então, a gente não tem muito incentivo para estar fazendo os passos todos certinho como seria dentro do hospital. Lá você vê o paciente todos os dias... vê se ele está evoluindo ou não, é fácil acompanhar, mas dentro da unidade básica é muito difícil. ” (E1)

“O relatório ainda eu faço. Mas aí tem outra coisa para fazer, aí eu não perco tempo em ficar procurando diagnóstico. Faço mais a parte de coleta de dados, o exame físico que eu nunca deixo de fazer, o que eu orientei o paciente. E se você for ver eu nunca completo o processo de enfermagem, né? ” (E8)

“Mas o que mais eu deixo deficiente é o diagnóstico de enfermagem, que não consta, porque histórico eu anoto, exame físico eu anoto. O que eu achei e as ações de enfermagem todas eu anoto. Então o que falta no meu registro é o diagnóstico. ” (E9)

“A única coisa que eu não consigo trabalhar até então é com a questão da metodologia nova de CIPE...CIAP...Há, a gente faz uma consulta de enfermagem, mas aí eu estou falando de mim. Não sei se as meninas fazem uma consulta de enfermagem, mas eu não uso essas coisas, não[...]” (E11)

“Em relação a aplicação total do PE no centro de saúde. Porque a gente consegue fazer uma avaliação do sujeito nas consultas agendadas, então no atendimento individual a gente consegue...é....fazer uma anamnese, né? Encontrar problemas para intervir, fazer intervenções pontuais, só que a gente não consegue uma avaliação periódica desse sujeito, por exemplo, a não ser que seja um caso muito grave que volte semanalmente[...]” (E12)

Os depoimentos mostram que os enfermeiros realizam diagnósticos, mesmo que não o nomeiem como tal ou reconheçam esse momento. Afinal, colhem dados e tomam decisões a partir dos dados colhidos. Eles confundem o uso de classificações com o raciocínio clínico que realizam diariamente, mas não registram. Na verdade, o enfermeiro não irá “perder tempo procurando um diagnóstico”, afinal a coleta de dados já o levou a pensar qual é a necessidade prioritária do paciente e quais intervenções implementar. O uso de classificações apenas o auxiliaria a nomear fenômenos de forma padronizada, comunicando isso a outros membros da equipe, o que facilitaria o

acompanhamento do paciente e daria visibilidade ao cuidado de enfermagem. Pode-se entender que os enfermeiros não se apropriaram do PE como um instrumento tecnológico[37], ou seja, não o veem como um modo organizado de expressar seu conhecimento cientifico, avaliando o PE apenas como um conjunto de leis com o qual se tem pouca aproximação.

A Taxonomia da Classificações Internacional das Práticas de Enfermagem (CIPE) e Classificação Internacional da Atenção Primária (CIAP) também foram citadas como possibilidades de aplicação dentro do DE como passo do PE, o que pode ser avaliado como um assunto de interesse dos enfermeiros de Campinas, mesmo não sendo abordados como elementos inseridos na legislação.

Outros relatos referem-se ao hospital como espaço de melhor “controle” do paciente, então maior facilidade de aplicar o PE, mostrando dificuldade de contextualiza-lo e aplica-lo na Atenção Primária. Pode-se supor que esse pensamento está relacionado aos primórdios da enfermagem, nascida com e para as atividades hospitalares, com o intuito de organiza-lo e diminuir as ações negativas do mesmo nos pacientes[36].

Associado ao aspecto que se refere ao nascimento da enfermagem, a formação acadêmica também tem seu papel na reprodução desse olhar para as práticas do enfermeiro, perpetuando um ensino mais voltado para a inserção do enfermeiro no hospital e a aplicação do PE melhor ensinada nesse espaço. Autores gaúchos também encontram relatos parecidos em estudo realizado na APS no Rio Grande do Sul[62].

Essa percepção dos enfermeiros que o PE no hospital é melhor aplicado ou mais fácil de fazê-lo advém ainda do olhar para o indivíduo como um ser portador de uma doença, de um problema ou uma deficiência. Esses enfermeiros afirmam que no ambiente hospitalar o cuidado ao paciente é mais previsível e controlado, porém, o enfermeiro hospitalar também realiza PE para pacientes que ele não conhece totalmente ou não acompanha diariamente, daí o valor dos registros, independente do contexto de assistência. Pode-se observar aqui uma abordagem do indivíduo pautado no modelo clinico, onde objeto de trabalho é o corpo doente[36;37] em associação ao estudo que discute a dificuldade da mudança de paradigma por parte da enfermagem, reproduzindo um olhar biomédico[56]. Assim a lente reducionista do olhar clinico e biomédico dificulta o entendimento do enfermeiro quanto ao indivíduo sadio que vive a sua vida, tem um trabalho, se insere numa família e numa comunidade.

O trabalho do enfermeiro na APS carrega em seu bojo a característica de relação com o outro, esse outro que na maioria das vezes não porta uma doença ou deficiência, mas está vivendo aspectos inerentes a algum momento de seu ciclo vital.

Esse descompasso pode se relacionar à medicalização dos corpos, bem como da percepção do PE e das ações dos enfermeiros. O que tem pouca aproximação coma perspectiva difundida na APS.

Dando continuidade a essa categoria, os enfermeiros também relatam, embora de forma indireta, confusão entre PE e SAE, que pode estar relacionado as mudanças da legislação do exercício profissional ocorridas nas últimas décadas, bem como a inserção de novas nomenclaturas, sendo SAE utilizada apenas no Brasil e inserção do termo PE no ano de 2000. Esta confusão de conceitos também pode estar associada a dificuldade de aplicação do PE. Seguem as falas que ilustram o exposto:

> *"Processo de enfermagem é toda readequação da equipe, toda reestruturação...que eu percebo assim... elaboramos todos os processos é....escala de salas, é como posso dizer...procedimentos e tudo mais. "* (E2)

> *De rotinas, de arrumar as salas, cada um na sua função. "* (E3)

> *"Bom aplicar SAE nós não aplicamos, isso porque nem tem como né...a não ser em acamados quando nós vamos fazer visita domiciliar[...]"* (E3)

> *"Falando aqui do centro de saúde...imagino que processo de enfermagem é a organização de tudo o que a enfermagem faz dentro da unidade, então é você seguir um processo e traçar metas, ter estratégias, é....avaliar até o que você fez de acordo com cada setor. Imagino que seja uma coisa muito ampla, então a gente ir dando passos, organizar determinadas coisas né? Por exemplo, uma coisa que a gente está tentando organizar agora aqui é o grupo do hiperdia...avaliação de hipertensos e diabéticos, outra coisa também que a gente já está fazendo um pouco é a questão das gestantes, a gente acho que tem até um controle legal sobre isso. "* (E7)

Os relatos expostos acima expressam uma fragilidade no tocante ao entendimento do que é PE, SAE, CE e Processo de Trabalho, essa confusão pode ser um desencadeador da pouca aplicação do PE na APS. Alguns estudos atuais utilizam PE como sinônimo de SAE[63], ou como sinônimo de CE[64], o que causa mais

confusão aos profissionais menos habituados aos conceitos de SAE e PE constantes na legislação atual.

Não se deve colocar toda a responsabilidade nesta confusão conceitual, mas a dificuldade de conceituar aquilo que se propõe a realizar, pode produzir um desserviço na condução da aplicação do PE em nível municipal, principalmente no contexto aqui estudado, em que não há homogeneidade nas orientações e práticas quanto a esta atividade.

Ao entendermos o PE como instrumento tecnológico, como exposto em outro momento nessa discussão, a confusão em sua conceituação diminui sua capacidade em atingir seu objetivo como modo de organizar o conhecimento no atendimento a pessoa, família ou comunidade e pode interferir na sua efetiva aplicação. Esta discussão poderia ter sido feita na categoria a seguir, ao olharmos todos esses temos como conjunto de conhecimentos, mas para fins didáticos foram abordados junto aos elementos internos que interferem na aplicação do PE.

5.2.3 O Saber do Enfermeiro

O saber emerge como categoria e pode ser analisado tanto como aspecto de cunho extrínseco, como de cunho intrínseco, por isso colocado como terceira categoria. Opta-se por não separar esses dois momentos de discussão por acreditar que desse modo pode ser melhor analisado. A partir dos discursos conceitua-se saber como conhecimento, qualificação, compreensão, entendimento, entre outros conceitos concernentes. Pode-se pontuar aqui que esse saber também envolve tecnologias diversas relatadas por Merhy[51], os enfermeiros, percebem o saber como um elemento importante à aplicação do PE e ao mesmo tempo acreditam ser deficiente desde a graduação, o que implica no uso incompleto e incorreto dessa tecnologia.

> *"Olha, para ser bem sincera, eu não tive uma formação boa nessa parte para processo de enfermagem. Hoje sendo profissional eu vejo quão ruim foi. Eu apliquei mais processo de enfermagem no hospital. É, talvez não como é a forma que eu entendo que é hoje, em centro de saúde mesmo. Na faculdade, se eu fiz dois, ah não, nem dois, foi bastante. Não tem uma formação boa.*

Depois que eu descobri o CIPESC, aqui mesmo na prefeitura, numa reunião ou outra. " (E8)

"No meu caso que me formei em 2004, a gente quando estudou processo de enfermagem a gente ficou focado muito no NANDA, né? E via as diferentes virtudes baseadas na NANDA que é essencialmente hospitalar. Então, a gente é um pouco carente nas opções que a gente tem, que não necessariamente que todas as vezes se aplicam nas especificidades da atenção básica. Acho que precisava melhorar um pouco o acesso a essa informação e trabalhar um pouco mais isso. " (E9)

"Agora eu estou com aluna aqui e eu percebo também, que mesmo a aluna de enfermagem não sabe. E falo: você não pode falar sobre...E elas: eu também não sei. " (E11)

"É ...é isso, eu acho que conta para mim, apesar de eu ser formada por uma faculdade voltada para a saúde pública é que esse conhecimento do processo de enfermagem na atenção primária veio depois da graduação, né? Não veio durante, então a gente é muito apegada e as formações dos universitários está muito vinculada ainda à área hospitalar. Então, a gente cai numa saúde pública. Eu vim da área hospitalar também, a pós-graduação foi na área hospitalar. A gente chega e quer tentar ter um raciocínio que a gente tinha na área hospitalar, sendo que aqui as influencias são muito maiores, então na minha visão o PE aqui teria mais potencial do que na área hospitalar, só que a gente não aplica. No meu caso inicial foi o desconhecimento, né? Porque eu vim conhecer o processo de enfermagem depois de quatro anos de graduada...na saúde pública. E depois do desconhecimento a minha não organização para por isso em pratica. " (E12)

Nas falas surgem elementos que se traduzem na compreensão de que o aprendizado deficiente sobre PE na graduação, mesmo para aplicação no hospital interferem em sua aplicação. Com relação à aplicação na APS apontam ainda maior despreparo, que ocorre desde a formação acadêmica. Estes resultados também foram encontrados no estudo de Krauzer[62].

Corroborando com esse resultado, um estudo realizado na Nigéria afirma que as enfermeiras clinicas daquele país tem um conhecimento limitado sobre PE,

interferindo na aplicação do mesmo e na implementação das taxonomias diagnósticas[64].

De acordo com os relatos dos entrevistados o ensino do PE está voltado à sua aplicação no hospital, mesmo em instituições de ensino que são consideradas “voltadas para a saúde pública”, podendo considerar como sinônimo de APS. Entretanto vale ressaltar que os hospitais também fazem parte do SUS, porém encontram-se em outro nível de complexidade do sistema de saúde.

Os relatos parecem desvelar desejo de aprender e usar o PE como instrumento de qualificação da prática profissional. Isso é especialmente estimulante quando a literatura traz que o ensino proporciona aumento de seu uso e melhora dos registros em todos os setores[65].

No caso de Campinas observa-se que ao mesmo tempo em que há um descontentamento quanto ao fato da instituição não dar melhores condições de trabalho, dar pouca ênfase e importância ao trabalho do enfermeiro e não proporcionar meios para instrumentaliza-los de forma homogênea e efetiva para a aplicação do PE, existe um o desejo de aprender e realizar o processo, bem como uma avaliação de que ele é importante para o trabalho do enfermeiro e para o cuidado, aqui é entendido como instrumento de trabalho do enfermeiro. As falas a seguir mostram esse resultado:

> *“Então o que eu falei. Acho assim, super bom para a qualificação profissional mesmo, isso que eu falei, ele acaba tendo muito mais autonomia porque a gente vai estar qualificando ele para isso, acho que o processo qualifica o profissional e aí ele vai ter mais autonomia para fazer a parte dele, é isso. ” (*E4)

> *“Então eu acho que é uma coisa que...é uma técnica que faz parte do nosso serviço e que a gente precisa se apropriar adequadamente dele e fazer direitinho para que a gente possa fazer um bom atendimento, porque se as coisas não forem bem fundamentadas igual é o processo eu acho que só vai complicando viu? ”* (E5)

> *“Bom a percepção que eu tenho dele...é....que um tanto dele ajuda bastante a vida dos enfermeiros a gente conseguir conduzir a saúde do paciente, né? [...]acho bacanérrimo, não acho que dá para o enfermeiro viver sem é a parte de coleta de dados, uma boa anamnese é fundamental, exame físico é fundamental[...] (E8)*

"Eu acho que a gente consegue fazer a sistematização dessa assistência a gente consegue fazer todas as etapas desse processo de enfermagem, no entanto a gente não consegue é documentar o que a gente faz. Não tem uma sistematização no registro desse processo de enfermagem[...]O processo é o todo, todas as etapas que a gente faz, sistematização mesmo é no sentido de a gente organizar o processo como um todo e registrar, então sistematização nesse sentido do dicionário mesmo de organizar o processo de enfermagem que a gente executa e registrar[...]Registro de enfermagem, a gente é falho no registro do processo como um todo. " (E9)

"É muito importante…a gente...é….principalmente para o enfermeiro, é exclusivamente para o enfermeiro, é necessário, meio que obrigação do enfermeiro está fazendo o processo de enfermagem. E qualifica mesmo...sistematiza o atendimento[...] (E10)

"Então acho que a gente está muito principiante em relação a aplicação de todas as etapas do processo, a gente não utiliza nenhuma metodologia para sistematizar isso, o que é ruim, né? " (E12)

O saber enquanto instrumento tecnológico não material[50] potencializa a ação dos enfermeiros na prestação do cuidado, e este aspecto é observado nas falas descritas acima. Os enfermeiros entendem que o PE qualifica a assistência, é necessário para o trabalho e exclusivo do enfermeiro, mostrando que há conhecimento deste como parte integrante da profissão mesmo que seja de difícil conceituação em algum momento ou que haja confusão com outros conceitos e nomenclaturas, corroboram com esse resultado os mesmos encontrados em alguns estudos[63;64;65].

Também explicita um dado importante que diz respeito ao fato dos enfermeiros compreenderem a diferença entre pensamento lógico com uso de conhecimento clínico e outros recursos cognitivos na atenção à saúde. Os enfermeiros alegam usar esse conhecimento mas possuem dificuldade do registro dessa ação e por isso alegam não realizar PE. Portanto a falha do registro pode suscitar uma percepção equivocada da inexistência de aplicação do PE, embora alguns enfermeiros relatem não realizar nem PE nem registro de suas ações e possuam dificuldade de compreensão do conceito deste.

6. CONCLUSÃO

Á partir dos relatos dos enfermeiros da APS, emergiram três categorias temáticas: “Situações extrínsecas que interferem na realização do PE”; “Situações intrínsecas que interferem na realização do PE” e “O saber do enfermeiro”.

Dentre as situações extrínsecas, verificou-se que os enfermeiros trazem menção a situações que não são especificas da aplicação do PE, mas compromete sua realização. Compreendem que a aplicação, ou não, do PE está relacionado a historicidade do trabalho da enfermagem, suas relações sociais, bem como suas interações com outras profissões em especial com o profissional médico cujo qual ainda apresenta uma disputa de poder pelo espaço institucional.

Nesta mesma vertente sentem-se cerceado em mudanças que deseja implementar, o que o desestimula, identifica excesso de trabalho, com multiplicidade de tarefas, além da escassez de recursos humanos, falta de investimento no setor saúde e em específico, na enfermagem. As narrativas desvelam a conexão da pouca aplicação do PE ao momento político, social e econômico, tanto da cidade de Campinas, quanto do País.

Ainda nesta categoria o COREN aparece nas falas como entidade fiscalizadora, que propulsiona a realizar o PE por obrigação legal sem uma aproximação com a profissão na APS.

Quanto às situações específicas do PE em si e que interferem em sua realização, os enfermeiros apontam um descompasso com a APS e as atribuições que os enfermeiros exercem nesta especialidade . Eles compreendem o PE como algo mais facilmente aplicável no contexto hospitalar, além disso, apresentam dificuldade de conceituar o que é PE, demonstrando constante confusão com Classificações de Enfermagem, fases especificas do PE, SAE, o que contribui para dificultar sua aplicação na APS.

Também compreendem pouco como o registro das ações executadas é valioso e dá visibilidade para o cuidado de enfermagem, como ciência ou como possibilidade de continuidade do cuidado ou pesquisas científicas.

Na terceira categoria, o “Saber do Enfermeiro” trouxe a compreensão dos enfermeiros quanto ao desconhecimento e despreparo para a realização do PE desde a graduação. Essa dificuldade, esse desconhecimento, também foi justificado pelo

aprendizado, quando houve, ter sido voltado à assistência hospitalar e percebido como saber diverso daquele aplicável à APS.

Apesar das situações externas e internas ao PE serem em sua maioria percepções negativas para a implementação deste, os enfermeiros o avaliam como importante e desejam realiza-lo em sua prática assistencial, porém também acreditam que, para que isso ocorra, é necessário maior investimento no profissional enfermeiro e melhores condições de trabalho e na área da saúde como um todo dentro da APS.

Assim, como fatores dificultadores surgem os de ordem política, econômica e social na interface com a área de saúde e causando interferência no trabalho do enfermeiro na APS.

A história da profissão também interfere na aplicação do PE a medida que o enfermeiro necessita ganhar espaço dentro das instituições e garantir a sua autonomia frente a outros profissionais.

A pouca aproximação dos enfermeiros com a legislação de enfermagem e pouca aproximação da legislação com a prática da enfermagem na APS também foi apontada como fator dificultador, associado a isso a pouca de compreensão dos conceitos do PE pela falta de contato com este, bem como devido ao fato de haver posições conflitantes quanto a definições deste na literatura.

Por fim, porém não menos importante, também emerge como fator dificultador a formação do enfermeiro, desde a academia, mostrando se falha no ensino do PE.

Quanto aos fatores facilitadores, surgiu apenas um que foi a avaliação de sua importância para a prática e qualificação profissional. Este dado está em consonância com as falas e percepções dos enfermeiros, sendo encontrados mais fatores dificultadores que facilitadores.

Portanto evidencia-se desafios à profissão, em especifico para os enfermeiros da APS quanto a aplicação do PE como a aproximação da legislação à prática da enfermagem na APS, o repensar o ensino do PE na graduação, e a compreensão da finalidade do trabalho na APS. Além é claro, de desmistificar a aplicação do PE no contexto da APS e a apropriação dos conceitos de PE, SAE e CE conforme preconiza a legislação, quiçá uniformizar esses conceitos para simplifica-la e facilitar seu entendimento e melhor aplicação do PE.

Esses desafios podem ser entendidos como potencialidades para aplicação do PE, desde que possibilitadas por ações baseadas numa política, no caso de Campinas, municipal de educação permanente visando incrementar o trabalho realizado,

instrumentalizando os enfermeiros para sua realidade e visibilizando a enfermagem como prática social num trabalho coletivo em saúde voltada ao SUS e à APS.

7. REFERÊNCIAS

1.Campinas (SP) Secretaria Municipal de Saúde de Campinas. Atenção à saúde/Protocolos 2014. (Acesso em 26 de maio de 2016). Disponível em: (http://www.campinas.sp.gov.br/saude/).

2.Silva EGC, Oliveira VC, Neves GBC, Guimarães TMR. O conhecimento do enfermeiro sobre a Sistematização da Assistência de Enfermagem: da teoria à prática [internet]. Rev Esc Enferm USP 2011; 45(6):1380-6. (citado em 05/04/2017) Disponivel em http://www.ee.usp.br/reeusp/.

3.Neves RS, Shimizu HE. Analise da implantação da Sistematização da Assistência de Enfermagem em uma Unidade de Reabilitação. Rev. Bras Enferm. 2010; 63 (2): 222-9.

4.Riva ,FJP, Martín-Igles S, del Cerro JLP, Arenas CM, López MG, Lagos MB. Reffectiveness of Nursing Process Use in Primary Care. International Journal of Nursing Knowledge, 2016; 27(1).

5.Abrantes R.M. Sistematização da Assistência de Enfermagem na ótica de enfermeiros de unidades de terapia intensiva. 2010. Dissertação de Mestrado, Centro de Ciências da Saúde, Universidade Federal da Paraíba, João Pessoa.

6.Dantas CN, Santos VEP, Tourinho FS. A Consulta de Enfermagem como Tecnologia do Cuidado à Luz dos Pensamentos de Bacon e GalimbertTexto Contexto Enferm, 2016; 25(1).

7.Pokorski S, Moraes MA, Chiarelli R, Constanzi AP. Processo de Enfermagem; da Literatura à prática. O que de fato estamos fazendo? Rev Latino-am Enfermagem. 2009; 17(3). (Acesso em junho, 2016). Disponível em: http://www.scielo.br/pdf/rlae/v17n3/pt_04.pdf.

8.Cavalcante, RB et al. Experiências de Sistematização da Assistência de Enfermagem no Brasil: um estudo bibliográfico. Revista de Enfermagem UFSM, Santa Maria, v.1, n.3, p 461-471, 2011.

9.Tornavall E, Wahren LK, Wilhelmsson S. Impact of primary care management on nursing documentation. Journal of Nursing Management, 2007, 15, 634–64.

10.Salvador TTCO, Santos VEP, Dantas CN. Caracterização das dissertações e teses brasileiras acerca da interface processo de enfermagem e atenção primária. Revista Mineira de Enfermagem. 2014; 18(2): 295-309.

11.Conselho Federal de Enfermagem (COFEN). Resolução COFEN 358/2009 [internet] Brasilia, DF, 2009. (citado em 26/05/2016). Disponível em www.cofen.gov.br.

12.Duarte MTC, Ayres JÁ, Simonetti JP. Consulta de Enfermagem: Estratégias de Cuidado ao Portador de Hanseníase em Atenção Primária [internet]. Texto Contexto Enferm. Florianópolis, 2009 Jan-Mar; 18(1): 100-7. (citada em 15/04/2017) Disponível em: http://www.scielo.br/pdf/tce/v18n1/v18n1a12.

13.Nogueira MJC. O processo de enfermagem em enfermagem comunitária: uma proposta. Rev Paul Enferm 1993; 3(1): 15-20 , apud. Duarte MTC, Ayres JA, Simonetti JP. Consulta de enfermagem ao portador de Hanseníase: proposta de um instrumento para aplicação do processo de enfermagem; Rev Bras Enferm, Brasília 2008; 61(esp):767-73.

14.Barros ALBL, Sanches CG, Lopes JL, Dell"Aqcua MCQ, Lopes MHBM, Genco e Silva, RC. Processo de Enfermagem: guia para a prática/COREN. São Paulo: COREN; 2015.

15.Garcia, TR et al. Integralidade da Atenção no SUS e Sistematização da Assistência de Enfermagem. Porto Alegre: Artmed; 2010.

16.Carvalho EM, Bachion MM, Dalri MCB, Jesus CAC.Obstáculos para a implementação do Processo de Enfermagem no Brasil. Rev enferm UFPE on line. 2007 jul./set.; 1(1):95-99.

17.Ribeiro GC. Diagnóstico Situacional da Sistematização da Assistência de Enfermagem em uma Unidade Básica de Saúde de Campinas-SP [Dissertação]. São Paulo: Universidade de São Paulo, 2015.

18.Tannure MC, Pinheiro, AM. SAE: Sistematização da Assistência de Enfermagem: guia prático. Rio de Janeiro: Guanabara Koogan; 2ª ed 2010.

19.Cianciarullo TI, Gualda DMR, Melleiro MM, Anabuki MH. Sistema de Assistência em Enfermagem: evolução e tendências. 5.ed. São Paulo: Ícone, 2012. p. 145-172.

20.Conselho Federal de Enfermagem (COFEN). Resolução COFEN 429/2012 [internet]. Rio de Janeiro(citado em 26/05/2016). Disponivel em www.cofen.gov.br.

21.Adami NP, Franco LHRO. Brêtas ACP, Ransan LMO, Pereira AL. Características básicas que diferenciam a consulta de enfermagem da consulta médica. Acta Paul Enferm. 1989; 2(1):9-13.

22.Conselho Federal de Enfermagem (COFEN). Decreto 7.498/86 [internet]. Rio de Janeiro (citado 30/04/2017). Disponível em (http://www.cofen.gov.br/lei-n-749886-de-25-de-junho-de-1986_4161.html.

23.Conselho Federal de Enfermagem (COFEN). Decreto 94.406/87 [internet]. (citado em 30/04/2017). Disponível em www.cofen.gov.br/decreto-n-9440687_4173.html.

24.Conselho Federal de Enfermagem (COFEN). Resolução n. 159/ 193 [internet]. (citado em 01/05/2017). Disponível em *www.cofen.gov.br/resoluo-cofen-1591993_4241.html.*

25.Conselho Federal de Enfermagem, Resolução COFEN 272/2000 [internet]. (citado em 03/05/2017). Disponível em *www.cofen.gov.br/resoluo-cofen-2722002.*

26.Carvalho G. A saúde pública no Brasil Estud. av. vol.27 (78) São Paulo; 2013.

27.Matta GC, Morosini MVG. Atenção Primária à Saúde: dicionário da Educação Profissional em saúde [internet] Brasilia, DF. (citado em 01/05/2017) disponível em: http://www.epsjv.fiocruz.br/dicionario/verbetes/ateprisau.html.

28.Brigadão JIM, Oliveira FM. O programa Paideia de Saúde da Família uma nova perspectiva no campo da produção da saúde. Cadernos de Gestão Pública e Cidadania, v10, n 47-jul/dezembro, 2005.

29.Marques D. O gênero e o trabalho em enfermagem na atenção básica: percepção das enfermeiras [tese]. São Paulo, Universidade de São Paulo, 2008.

30.Nascimento EPL, Correa CRS, Nozawa MR. História as saúde pública em Campinas [internet] Campinas 2010. (citado em 02/05/2017). Disponível em www2.ibfc.org.br/concurso/...campinas.../historia-da-saude-publica-em-campinas.pdf.

31.Barreira, IA. A enfermeira Ana Nery no pais do Futuro: a aventura da luta contra a tuberculose[tese]. Rio de Janeiro, Escola de Enfermagem Ana Nery, Universidade Federal do Rio de Janeiro, 1992.

32.Villa TCS, Mishima SM, Rocha SMM. A enfermagem nos serviços de saúde pública do estado de São Paulo. In: Almeida MCP, Rocha SMM (organizadores). O trabalho de enfermagem. São Paulo; Cortez, 1997. P.27-60.

33. Brondani Jr. DA Davi, Heck RM, Ceolin T, Viegas CRS. Atividades gerenciais do enfermeiro na estratégia de saúde da família. R.Enferm.USFM 2011 jan/abr;1(1): 41-50.

34. Jensen R, Cruz DALM, Tesoro MG, Lopes MHBM. Tradução e adaptação cultural para o Brasil do Modelo Developing Nurses “Thinking”. Rev Latino-Am Enfermagem. 2014; 22(2):197-203.

35. Gerk MAS, Barros SMO. Intervenção de Enfermagem para o diagnóstico de enfermagem mais frequentes em dois serviços públicos de assistência a saúde da mulher. Acta Paulista de Enfermagem. 2005; 18(3): 260-8.

36.Almeida MCP, Rocha SMM. Considerações da enfermagem enquanto trabalho. In: Almeida MCP, Rocha SMM (Orgs). O Trabalho em Enfermagem, São Paulo: Cortez, 1997; cap.1, p. 15-26.

37.Mendes Gonçalves RB. Práticas de Saúde: processos de trabalho e necessidades. São Paulo: CEFOR, 1992.

38.Creswell JW. Investigação Qualitativa e Projeto de pesquisa: escolhendo entre cinco abordagens. 3.ed. Porto Alegre: Editora Penso; 2014.

39.Minayo MCS. O desafio do conhecimento: Pesquisa Qualitativa em Saúde. 14ª ed.; são Paulo: HUCITEC, 2014.

40.Campinas (SP) História de Campinas [internet]. (citado em 06 de maio de 2017). Disponível em : (http://www.campinas.sp.gov.br/sobre-campinas/campinas.php.

41.IBGE. Panorama da cidade de Campinas [internet]. (citado em 05/05/2017). Disponível em: http://www.cidades.ibge.gov.br/v4/brasil/sp/campinas/panorama..

42. L'abbate S. O direito à saúde: da reivindicação à realização. Projetos de política de saúde em Campinas. [tese]. Campinas: Universidade Estadual de Campinas; 1990.

43. Minayo MCS. Analise qualitativa: teoria, passos e fidedignidade. Ciência & Saúde Coletiva. 2012; 17(3); 621-626.

44.Miguel FVC, Revistaodisseia-PPgEL/UFRN, nº 5 (jan-jun), 2010 I S S N 1 9 8 3 - 2 4 3 5.

45.Belei SR, Gimenez-Paschoal EM, Nascimento EN, Matsumoto PHVR. O uso de entrevista, observação e videogravação em pesquisa qualitativa. Cadernos de Educação. 2008; 30: 187 – 199.

46.Marx K. Manuscritos econômico-filosóficos. São Paulo: Martin Claret, 2002. 198p.

47.International Council of Nurses (ICN). Notas sobre a enfermagem: um guia para cuidadores na atualidade. Tradução Telma Ribeiro Garcia. Rio de Janeiro: Elsevier, 2010.

48.Rocha SMM, Almeida MCP. O processo de trabalho da enfermagem em saúde coletiva e a interdisciplinaridade. Rev latino-am Enfermagem - Ribeirão Preto, 2000; 8(6): 96-101.

49.Mendes Gonçalves RB. Tecnologia e organização social das práticas de saúde: características tecnológicas do processo de trabalho na rede estadual de centros de saúde de São Paulo. São Paulo: HUCITEC/Abrasco, 1994.

50.Almeida MCP, Rocha JSY. O saber de Enfermagem e sua dimensão prática. São Paulo: Cortez, 1986.

51.Merhy, EE; Franco, TB; Trabalho, produção do cuidado e subjetividade em saúde textos reunidos,ed Hucitec, SP, 2013.

52.Machado MH, Aguiar Filho W, Lacerda WF, Oliveira E, Lemos W, Wermelinger M, Vieira M, Santos RM, Souza Junior PB, Justino E, Barbosa C. Enferm. Foco 2016; 7 (ESP).

53.Conselho Federal de Enfermagem (COFEN). Pesquisa Perfil da Enfermagem no Brasil - 2013. [Internet]. (Citado em 03/05/2017). Disponível emhttp://www.coren-sp.gov.br/node/40505 perfil da enf são paulo.

54.Santos AS, Traldi MC. Administração de enfermagem em saúde coletiva. Barueri: Manole; 2015 403p. (Serie Enfermagem e saúde; vol. 5)

55.Marques D. A trajetória do Programa de Saúde da Familia em Campinas e a contribuição da enfermagem [dissertação]. Campinas, Faculdade de Ciências Médicas, Universidade Estadual de Campinas, 2003.

56.Takemoto MLS. O trabalho de enfermagem em unidades básicas de saúde e a proposta de mudança do modelo de atenção a saúde no Município de Campinas-SP. [Dissertação] Campinas, Faculdade de Ciências Médicas, Universidade Estadual de de Campinas, 2005.

57.Kempfer SS, Birolo IVB, Meirelles BHS, Erdmann AL. Reflexão sobre um modelo de sistema organizacional de cuidado de enfermagem centrado nas melhores práticas. Rev Gaúcha Enferm., Porto Alegre (RS) 2010 set;31(3):562.

58.Merhy EE. Saúde e direitos: tensões de um SUS em disputa, molecularidades. Saúde Soc [serial on the internet]. 2012 [cited 2013 Dec 18];21(2):267-79. Disponível em: http:// www.scielo.br/pdf/sausoc/v21n2/a02v21n2.pdf.

59.Bossato HRA. Política de Financiamento do SUS no Conselho Municipal de Saúde de Niterói: Uma abordagem sócio-histórico [Dissertação]. Niterói. Escola de Enfermagem Aurora Afonso, Universidade Federal Fluminense, 2011.

60.Marques D, Silva EM. A enfermagem e o Programa Saúde da Família: uma parceria de sucesso? Rev Bras de Enferm. 2004; 57(5); p. 545-550.

61.Takemoto MLS, Silva EM. Acolhimento e transformações no processo de trabalho de enfermagem em unidades básicas de saúde de Campinas, São Paulo, Brasil Cad. Saúde Pública, Rio de Janeiro, 23(2):331-340, fev, 2007.

62.Krauzer IM, Adamy EK, Ascari RA, Ferraz L, Trindade LL, Neiss M. Sistematização da Assitencia de Enfermagem na Atenção Básica: O que dizem os enfermeiros? [interent] Ciencia y Enfermeria XXI (2):31-38,2015. (citada em 28/05/2017) Disponível em: pesquisa.bvsalud.org/enfermagem/resource/pt/lil-764008.

63.Campos RMC, Ribeiro CA, Silva CV, Saparolli ECL. Consulta de Enfermagem em puericultura: a vivencia do enfermeiro na Estratégia de Saúde da Familia [internet].

Citada em 28/05/2017) Rev Esc Enferm USP 2011; 45(3):566-74 Disponível em: www.scielo.br/pdf/reeusp/v45n3/v45n3a03.pdf.

64.Momoh MA, Chukwu DO. Factors that militate against the use of nursing process: A hospital-based study. Continental Journal of Pharmaceutical Sciences (4) 6-9, 2010.

65.Odutayo PO, Olaogun AA, Oluwatosin AO, Ogunfowokan AA. Impact of an Educational Program on the use of Standardized Nursing Languages for Nursing Documentation Among Public Healt Nurses in Nigeria. NANDA Internacional Journal of Nursing Knowledge, Volume 24 (2), June 2013.

APÊNDICES

Apêndice 1

PROJETO DE PESQUISA: PROCESSO DE ENFERMAGEM NA ATENÇÃO PRIMÁRIA À SAÚDE: PERCEPÇÃO DOS ENFERMEIROS DE CAMPINAS/SP.

Roteiro de entrevista

Formulário número: ________

Parte 1: **Caracterização dos Participantes**

Dados Pessoais

Sexo ( ) feminino ( ) masculino
Idade: __________(anos)

Formação Profissional

( ) Graduação ( ) Especialização ( ) Mestrado ( ) Doutorado

( ) Outros: _________________________

Ano em que concluiu o curso de graduação em enfermagem: ________

Ano de admissão na Prefeitura Municipal de Campinas como enfermeiro: _______

Tempo de trabalho nesta unidade de saúde:______________

Parte 2: **Percepções dos enfermeiros acerca do processo de enfermagem**

1 – Fale-me sobre sua experiência quanto ao Processo de enfermagem na atenção primária.

2– Como você percebe o Processo de Enfermagem para a qualificação profissional?

3 - Como você definiria Processo de Enfermagem?

Apêndice 2

TERMO DE CONSENTIMENTO LIVRE E ESCLARECIDO

Título do projeto: **Processo de Enfermagem na Atenção Primária: Percepção dos Enfermeiros de Campinas/SP**

Pesquisador: Marta Patrícia Spazapan

Orientador: Profa. Dra. Elenice Valentim Carmona

Número do CAAE: 61606316.0.0000.5404

Caro participante,

Você está sendo convidado a participar como voluntário de uma pesquisa. Este documento, chamado Termo de Consentimento Livre e Esclarecido, visa assegurar seus direitos como participante e é elaborado em duas vias, uma que deverá ficar com você e outra com o pesquisador.

Por favor, leia com atenção e calma, aproveitando para esclarecer suas dúvidas. Se houver perguntas antes ou mesmo depois de assiná-lo, você poderá esclarecê-las com o pesquisador. Se preferir, pode levar este Termo para casa e consultar seus familiares ou outras pessoas antes de decidir participar. Se você não quiser participar ou retirar sua autorização, a qualquer momento, não haverá qualquer tipo de penalização ou prejuízo.

Justificativa e objetivos:

Este estudo tem como objetivo analisar as percepções dos enfermeiros a respeito do Processo de Enfermagem na atenção Primária da Secretaria Municipal de Saúde de Campinas. Seu desenvolvimento justifica-se por oferecer subsídios para repensar a implementação do Processo de Enfermagem na atuação do enfermeiro na atenção primária, o que tem repercussões para a qualificação da profissão como um todo.

Procedimentos:

Sua participação, voluntaria, significará ser entrevistada individualmente, em entrevista única e agendada, em local de sua escolha, com duração aproximada de 30 minutos. Quanto à gravação da entrevista em áudio, solicito que você coloque sua rubrica no espaço entre parênteses que corresponde ao que decidir, esclarecendo que, caso você não autorize a gravação, será excluído do estudo:

(_______________)autorizo que minha entrevista seja gravada; (_______________)não autorizo que a minha entrevista seja gravada.

Você tem a liberdade de se recusar a participar do estudo e mesmo que aceite, poderá retirar seu consentimento a qualquer momento, sem necessidade de apresentar justificativas.

Sigilo e Privacidade:

As informações coletadas são de caráter confidencial e sua identidade será mantida em sigilo de qualquer pessoa que não faça parte da equipe desta pesquisa. Sua identidade não será divulgada em nenhuma fase da pesquisa ou na divulgação dos resultados da mesma. Os dados colhidos serão armazenados em arquivos digitais, protegidos por senha, aos quais só a pesquisadora e orientadora terão acesso. Serão armazenados durante o desenvolvimento do estudo e destruídos após 60 meses.

Ressarcimento e Indenização:

Você não receberá qualquer valor em dinheiro pela sua participação, assim como não haverá qualquer despesa decorrente dessa participação, visto que a pesquisadora irá se deslocar até o participante para realizar a pesquisa. Entretanto, você terá a garantia ao direito a indenização diante de eventuais danos decorrentes de sua participação na pesquisa, previsto ou não neste TCLE, por parte do pesquisador e instituições envolvidas. Cabe enfatizar que o direito a indenização está prevista pelo Código Civil.

Desconfortos e riscos:

Esta pesquisa não apresenta riscos previsíveis, mas poderá haver desconforto com o tempo dispendido ao longo da entrevista, sendo que a pesquisadora compromete a minimizar esta ocorrência lhe deixando à vontade para terminar a entrevista quando você julgar necessário.

Benefícios:

Não haverá benefícios imediatos na sua participação no estudo, porem o benefício esperado é a contribuição para o conhecimento e potenciais melhorias nas práticas de enfermagem.

Contato:

Em caso de dúvidas sobre o estudo, você poderá entrar em contato com o pesquisador: Marta P. Spazapan, Rua Prof. Dr. Emilio Coelho, 546, Village-Campinas, fone (19) 32874210, cel.(19) 99741-4431, email mpspazapan@yahoo.com.br .

Em caso de denúncias ou reclamações sobre sua participação e sobre questões éticas do estudo, você pode entrar em contato com a secretaria do Comitê de Ética em Pesquisa (CEP) da UNICAMP das 08:30hs às 13:30hs e das 13:00hs as 17:00hs, na Rua: Tessália Vieira de Camargo, 126; CEP 13083-887 Campinas – SP; telefone (19) 3521-8936; fax (19) 3521-7187; e-mail: cep@fcm.unicamp.br

O Comitê de Ética em Pesquisa (CEP).

O papel do Comitê de Ética em Pesquisa (CEP) é avaliar e acompanhar os aspectos éticos de todas as pesquisas envolvendo seres humanos. A Comissão Nacional de Ética em Pesquisa (CONEP), tem por objetivo desenvolver a

regulamentação sobre proteção dos seres humanos envolvidos nas pesquisas. Desempenha um papel coordenador da rede de Comitês de Ética em Pesquisa (CEPs) das instituições, além de assumir a função de órgão consultor na área de ética em pesquisas.

Consentimento livre e esclarecido:

Após ter recebido esclarecimentos sobre a natureza da pesquisa, seus objetivos, métodos, benefícios previstos, potenciais riscos e o incômodo que esta possa acarretar, aceito participar e declaro estar recebendo uma via original deste documento assinada pelo pesquisador e por mim, estando todas as folhas rubricadas por nós:

Nome do(a) participante:____

Contato telefônico:____

E mail(opcional)____

____Data: ____/_____/______.
(Assinatura do participante)

Responsabilidade do Pesquisador:

Asseguro ter cumprido as exigências da resolução 466/2012 CNS/MS e complementares na elaboração do protocolo e na obtenção deste Termo de Consentimento Livre e Esclarecido. Asseguro, também, ter explicado e fornecido uma via deste documento ao participante. Informo que o estudo foi aprovado pelo CEP perante o qual o projeto foi apresentado. Comprometo-me a utilizar o material e os dados obtidos nesta pesquisa exclusivamente para as finalidades previstas neste documento ou conforme o consentimento dado pelo participante.

____Data: ____/_____/______.
(Assinatura do pesquisador

Anexo A

PREFEITURA MUNICIPAL DE CAMPINAS
Secretaria Municipal de Saúde

SUS

AUTORIZAÇÃO

056/2016

Autorizo a realização da Pesquisa intitulada **"PROCESSO DE ENFERMAGEM NA ATENÇÃO PRIMÁRIA: PERCEPÇÃO DE ENFERMEIROS DE UNIDADES BÁSICAS DE CAMPINAS-SP"**, que tem por objetivos: a) analisar as percepções das enfermeiras a respeito do Processo de Enfermagem na Atenção Primária da Secretaria Municipal de Saúde de Campinas e b) compreender a pouca adesão a implantação do Processo de Enfermagem na Atenção Primária.

Declaro estar ciente que a Pesquisa será desenvolvida por estudante do Programa de Pós-Graduação em Enfermagem, nível mestrado, na Faculdade de Enfermagem da Universidade Estadual de Campinas - Unicamp, junto a profissionais enfermeiros que atuam na atenção primária, em Centros de Saúde localizados na região do Distrito de Saúde Leste, sob a orientação da Professora Doutora Elenice Valentin Carmona.

Campinas, 20 de setembro de 2016.

Cármino Antonio de Souza
Secretário Municipal de Saúde
Matrícula: 125.344-1
CPF: 723.931.818-49

Anexo B

PARECER CONSUBSTANCIADO DO CEP

UNICAMP - FACULDADE DE CIÊNCIAS MÉDICAS DA UNIVERSIDADE DE CAMPINAS

DADOS DO PROJETO DE PESQUISA
Título da Pesquisa: PROCESSO DE ENFERMAGEM NA ATENÇÃO PRIMÁRIA: PERCEPÇÃO DE ENFERMEIROS DE CAMPINAS-SP
Pesquisador: MARTA PATRICIA SPAZAPAN **Área Temática:**
Versão: 2
CAAE: 61606316.0.0000.5404
Instituição Proponente: FACULDADE DE ENFERMAGEM DA UNICAMP
Patrocinador Principal: Financiamento Próprio
DADOS DO PARECER

Número do Parecer:

1.885.225

Apresentação do Projeto:
Este projeto de pesquisa teve como motivação minhas inquietações frente às notificações sobre Processo de Enfermagem (PE), advindas de visitas realizadas pelo Conselho Regional de Enfermagem (COREN) – subseção Campinas a várias unidades de saúde da Secretaria Municipal de Saúde (SMS) da Prefeitura Municipal de Campinas (PMC) nos anos de 2013 e 2014. Estas inquietações se deram após observar a heterogeneidade na realização do PE por parte dos enfermeiros da atenção primaria, assim como na compreensão das definições deste, ao que também me incluo. Tal falta de homogeneidade não se refere apenas aos enfermeiros da SMS da Prefeitura de Campinas, pois muitos fatores interferem na realização do PE, o que abrange desde o preparo na graduação até as políticas institucionais favoráveis ou não à sua implementação (Neves, Shimizu, 2010). A implantação do PE ainda ocorre de forma incipiente e fragmentada, mesmo sendo objeto de reflexão desde a década de 70, com os estudos de Wanda Horta (Abrantes, 2010). Como as visitas do COREN se deram em todos os Distritos de Saúde da SMS, houve uma mobilização dos diversos níveis hierárquicos da Secretaria no sentido de apropriar-se das definições e conceitos relacionados ao PE e iniciar discussões, bem como capacitações sobre esse tema tão pertinente à pratica do cuidado de Enfermagem. Desde então, surgiram grupos de discussão que tiveram como produto o desenvolvimento de protocolos, atualização de protocolos.

Endereço: Rua Tessália Vieira de Camargo, 126
Bairro: Barão Geraldo **CEP:** 13.083-887
UF: SP **Município:** CAMPINAS
Telefone: (19)3521-8936 **Fax:** (19)3521-7187 **E-mail:** cep@fcm.unicamp.br

anteriores, desenvolvimento do Regimento Interno de Enfermagem e de Procedimentos Operacionais Padrão - POP (Campinas,2016). Isto com intuito de instrumentalizar enfermeiros e fomentar conhecimento para implantação do PE na atenção primária. Este processo também aconteceu de maneira heterogênea em cada Distrito e Unidade Básica de Saúde (UBS). Ainda assim, reconhecendo em cada espaço um contexto diferente, faz-se imprescindível salientar que o trabalho iniciado promoveu avanço para a Enfermagem de Campinas e visibilidade da profissão. A referida cidade possui grande potencial por ser importante polo de universidades e centro industrial e tecnológico (Campinas, 2016). Também oferece campo de estagio para faculdades e cursos técnicos de enfermagem, influenciando o processo de aprendizagem dos novos profissionais. Atualmente a SMS conta com mais de 300 enfermeiros na atenção primaria, número bastante expressivo, se considerarmos este contingente como sujeitos históricos da enfermagem, profissão de um saber técnico e cientifico estruturado. Assim buscar entender, apreender, capturar quais são as percepções que estes sujeitos possuem sobre PE pode oferecer subsídios para novas estratégias no sentido de aprimorar a qualidade da assistência e incentivar a autonomia profissional.1.1.Processo de Enfermagem: contextualização e etapas PE é um instrumento metodológico que orienta o cuidado profissional de Enfermagem e a documentação da prática, aumentando a visibilidade e o reconhecimento da profissão. Quando realizado em instituições prestadores de serviços ambulatoriais de saúde, corresponde ao usualmente denominado nesses ambientes como Consulta de Enfermagem (COFEN, 2009). Trata-se de um processo dinâmico voltado para as ações do cuidado na prática profissional, visando identificar problemas de saúde, planejar, implementar ações e avaliar os resultados. Também pode ser definido como uma ferramenta intelectual da prática profissional do enfermeiro, que direciona a lógica clínica e as decisões no que tange a diagnósticos, intervenções, resultados e avaliações. Um modelo de pensamento e atuação que é documentado (Barros, 2015). A ideia de PE é antiga e surge com a enfermagem moderna, quando Florence Nightingale acredita que é necessário que as enfermeiras devam ser ensinadas a usar a observação e julgamento na sua pratica (COREN, 2015).

Porém, foi só na década de 50 que o termo PE foi introduzido na linguagem profissional pelas enfermeiras norte-americanas. Isto porque reconheciam que desenvolver um corpo de conhecimento era vital para a sobrevivência e evolução da profissão, tendo como objetivo dar sustentação cientifica ao cuidado, com conhecimento próprio, dinâmico e com várias formas de expressão. O desenvolvimento de Teorias de Enfermagem também se deu neste momento histórico e com o mesmo objetivo (Garcia, 2010). As Teorias de Enfermagem servem de referencial para análise da prática profissional, explicando de várias formas o modo de fazer Enfermagem. Tais Teorias são

Estas etapas são mencionadas a seguir (COFEN 2009):I – Coleta de Dados de Enfermagem (ou Histórico de Enfermagem): processo deliberado, sistemático e continuo, realizado com o auxílio de métodos e técnicas variadas, que tem por finalidade a obtenção de informações (significativas para o raciocínio clinico do profissional) sobre a pessoa, família ou a coletividade humana quanto a suas respostas em um dado momento do processo de saúde/doença ou processo de vida, que tornam possível a identificação de seus problemas ou potencialidades;II– Diagnostico de Enfermagem: processo de interpretação e agrupamento dos dados coletados na primeira etapa, que culmina com a tomada de decisão sobre os conceitos diagnósticos de enfermagem que representam, com mais exatidão, as respostas da pessoa, família ou coletividade humana em um dado momento do processo de saúde/doença ou processo de vida, e que constitui a base para a seleção das ações ou intervenções, com os quais se compostas por conceitos e definições que descrevem fenômenos, correlacionam e explicam situações, preveem acontecimentos e controlam resultados obtidos por meio das ações de Enfermagem (Tannure, 2008). É neste conjunto de conhecimentos que se localiza o PE, sendo ações executadas pelos profissionais de enfermagem, tendo em vista as necessidades da pessoa/família/coletividade que demandam cuidado profissional para solução de problemas de forma deliberada. O que exige habilidades cognitivas, técnicas e de relação interpessoal (Garcia, 2010). No Brasil, a pioneira em abordar o tema “Teoria de Enfermagem” e PE foi Wanda de Aguiar Horta, entre as décadas de 60 e 70: a princípio divulgando o conhecimento das enfermeiras norte-americanas e, posteriormente, divulgando o seu próprio, por meio da Teoria das Necessidades Humanas Básicas, inspiradas em Maslow (Garcia, 2010). Atualmente, o PE tem sido aplicado e estudado no Brasil e no mundo (Barros e Lopes, 2010). No Brasil também utilizamos o termo “Sistematização da Assistência de Enfermagem” (SAE), o que é bastante particular da regulamentação da enfermagem brasileira. Assim, verifica-se que existem algumas discussões e estudos que abordam SAE e PE de forma conflituosa, sendo que muitos autores mencionam “processo de enfermagem”, “metodologia da assistência de enfermagem” e a própria “sistematização da assistência de enfermagem” como sinônimos e, por vezes, com definições equivocadas. O conflito de definições dos termos também pode ser considerado um dificultador no entendimento e consequente aplicação destes na prática profissional (Ribeiro, 2015). Para o presente estudo, SAE e PE são distintos: PE foi definido acima, enquanto que a SAE é algo que organiza o trabalho profissional quanto ao método, pessoas e instrumentos, tornado possível a implementação do PE (COFEN 2009). Embora seja um processo dinâmico nem sempre linear, para fins didáticos e de compreensão, a Resolução COFEN 358/2009 afirma que o PE é organizado em cinco etapas subsequentes e interrelacionadas.

objetiva alcançar os resultados esperados;III – Planejamento de Enfermagem: determinação dos resultados que se espera alcançar e das ações ou intervenções de enfermagem que serão realizados para tanto, frente às respostas da pessoa, família ou coletividade humana em um dado momento do processo de saúde/doença ou processo de vida, identificadas na etapa de Diagnóstico de Enfermagem (DE);IV – Implementação: realização das ações ou intervenções determinadas na etapa de planejamento;V – Avaliação de Enfermagem: processo deliberado, sistemático e continuo de verificação de mudanças nas respostas da pessoa, família ou coletividade humana em um dado momento do processo de saúde/doença ou processo de vida, para determinar se as ações ou intervenções de enfermagem alcançaram o resultado esperado, e de verificação da necessidade de mudança ou adaptações nas etapas do PE. O PE é privativo do enfermeiro, cabendo a ele a liderança na sua execução e avaliação em todos os ambientes onde ocorra o cuidado de enfermagem (Resolução COFEN, 358/2009). Também é responsabilidade e dever dos profissionais da Enfermagem registrar, no prontuário do paciente e em outros documentos próprios, os dados coletados para assegurar reavaliações da assistência prestada, bem como a continuidade do cuidado, o que melhora a qualidade deste (Resolução COFEN 429/2012). Entretanto, na prática clínica existem dificuldades na implantação e utilização do PE no Brasil e em outros países, com falhas nos registros de uma ou mais fases do PE (Pokorski, 2009; Cavalcanti, 2011). Aparentemente, as mudanças de modelo assistencial que vem ocorrendo nas últimas décadas, focandose a atenção básica e mais precisamente a Saúde da Família e Comunidade, não tem influenciado de forma positiva a implantação do PE na atenção primária. As experiências de implantação do PE no Brasil são prioritariamente em ambiente hospitalar, nos níveis de atenção secundário e terciário, ocorrendo de forma muito incipiente na atenção primária (Cavalcante, 2011). As teses e dissertações desenvolvidas a respeito de PE na atenção primária são em pequeno número (Salvador; Santos; Dantas, 2014)

UNICAMP - FACULDADE DE CIÊNCIAS MÉDICAS DA UNIVERSIDADE DE CAMPINAS

Continuação do Parecer: 1.885.225

Este contexto demanda investigações para entender como favorecer tal implantação, visto que o desenvolvimento do PE contribui para o pensamento crítico, aperfeiçoa, responsabiliza e aumenta a satisfação profissional, além de qualificar a assistência. Assim, torna a pratica da profissão mais visível, legitima e autônoma, o que possibilita melhorar a qualidade do cuidado, permitindo ao enfermeiro sistematizar suas ações e delegar tarefas à equipe de enfermagem de forma clara e organizada (Gerk e Barros, 2005). Neste contexto, o pensamento crítico se faz cada vez mais necessário ao enfermeiro devido às rápidas mudanças relativas à atenção à saúde e a crescente complexidade do sistema de saúde. Assim, este estilo de pensamento envolve habilidades e atitudes para o raciocínio clínico que interferem nas ações e decisões assistenciais do enfermeiro (Jensen, 2014). A experiência clínica

remete a pensar que ainda temos pouca adesão à implantação do PE na atenção primária, o que se expressa por meio da atuação coerente dos enfermeiros quanto às necessidades dos pacientes, mas de forma não sistematizada e sem os registros pertinentes. O que vem acontecendo, apesar de inúmeros avanços e situações que deveriam facilitar a implantação: a evolução da Enfermagem como profissão, com uma prática organizada e cientifica, inserida em um modelo assistencial que é voltado para a atenção primária; o fato do PE ter legislação em Conselho de classe e função social; bem como o reconhecido valor das informações que são trocadas entre enfermeiro e paciente, relacionadas ao encaminhamento da efetivação do cuidado. Desta forma, o desenvolvimento de um estudo sobre as percepções dos enfermeiros da atenção primária quanto ao PE pode dar luz à compreensão da pouca adesão a ele na prática clínica e suscitar estratégias futuras que modifiquem este cenário. METODOLOGIA PROPOSTA: Trata-se de um estudo de abordagem qualitativa, que se caracteriza como reflexiva e interpretativa, pois possibilita considerar múltiplas perspectivas dos participantes, identificando variáveis não mensuráveis facilmente, permitindo escutar e minimizar as relações de poder. Este método possibilita ainda compreender o contexto em que os participantes do estudo abordam a questão/problema (Creswell, 2014).

Endereço: Rua Tessália Vieira de Camargo, 126
Bairro: Barão Geraldo **CEP:** 13.083-887
UF: SP **Município:** CAMPINAS
Telefone: (19)3521-8936 **Fax:** (19)3521-7187 **E-mail:** cep@fcm.unicamp.br

UNICAMP - FACULDADE DE CIÊNCIAS MÉDICAS DA UNIVERSIDADE DE CAMPINAS

Continuação do Parecer: 1.885.225

As matérias primas da pesquisa qualitativa compõem-se por um conjunto de substantivos que possuem sentidos complementares como experiência, vivência, senso comum e ação. Sua análise baseia-se no compreender, interpretar e dialetizar (Minayo, 2011). Também para esta autora o verbo principal da análise qualitativa é o “compreender” e na busca da compreensão é preciso exercitar o entendimento das contradições e conflitos na ação e linguagem pelos efeitos do poder, das relações sociais e dos interesses (Minayio, 2011). Pelo descrito acima o método qualitativo se mostra adequado e atende aos objetivos desta pesquisa que busca as percepções dos enfermeiros quanto ao PE. Os dados serão colhidos por meio de entrevista semi estruturada junto a enfermeiros da atenção primária. A questão norteadora será: Fale me sobre sua experiência quanto ao Processo de Enfermagem na Atenção Primária. Os dados serão analisados por meio de Analise de Conteúdo proposta por Bardin. Critério de Inclusão: Prevê-se como sujeitos deste estudo os 30 enfermeiros das Unidades Básicas de Saúde do Distrito de Saúde Leste da SMS de Campinas/SP Critério de Exclusão: Serão excluídos: enfermeiros com cargo gerencial; enfermeiros afastados no período de coleta de dados devido a férias ou licenças e enfermeiros que se recusarem a ter a entrevista gravada em áudio. METODOLOGIA DE ANÁLISE DE DADOS: Os relatos serão transcritos integralmente, considerando-se as falas, as pausas e os detalhes. Após a transcrição, o material será submetido à “Análise do Conteúdo Temático”, preconizada por Bardin (2011), o que contempla: pré-análise (leitura flutuante, exaustiva, representativa, homogênea e

pertinente ao objetivo de estudo); a exploração do material (codificação dos dados, identificação de categorias) e tratamento dos resultados. Nesta fase, os dados serão organizados em categorias, analisados e interpretados segundo a literatura pertinente. A pesquisadora e a orientadora farão, em separado, leituras atentas e criteriosas de cada um dos depoimentos, primeiramente buscando identificar o sentido global das percepções das enfermeiras, depois as categorias e discutirão os dados para o consenso

Objetivo da Pesquisa:

Analisar a percepção de enfermeiros a respeito do Processo de Enfermagem na atenção Primária da Secretaria Municipal de Saúde de Campinas.

Avaliação dos Riscos e Benefícios:

Riscos e benefícios segundo a pesquisadora:

Riscos: Não existem riscos previsíveis, mas poderá haver desconforto com o tempo dispendido ao longo da entrevista e/ou oficina, sendo que a pesquisadora compromete a minimizar esta ocorrência.

Benefícios: Não haverá benefícios imediatos na sua participação no estudo, porem o benefício esperado é a contribuição para o conhecimento e potenciais melhorias nas práticas de enfermagem.

Endereço: Rua Tessália Vieira de Camargo, 126
Bairro: Barão Geraldo **CEP:** 13.083-887
UF: SP **Município:** CAMPINAS
Telefone: (19)3521-8936 **Fax:** (19)3521-7187 **E-mail:** cep@fcm.unicamp.br

Comentários e Considerações sobre a Pesquisa:

Trata-se de um projeto de pesquisa de mestrado da aluna MARTA PATRÍCIA SPAZAPAN da Faculdade de Enfermagem da Universidade Estadual de Campinas (UNICAMP). Realizar-se-á uma pesquisa qualitativa em que será utilizada a técnica de entrevista semiestruturada em que serão convidados a participar trinta enfermeiros das Unidades Básicas de Saúde do Distrito de Saúde Leste da Secretaria Municipal de Campinas/SP. As entrevistas serão gravadas e posteriormente transcritas. Após a transcrição, o material será submetido à "Análise do Conteúdo Temático", preconizada por Bardin.

Financiamento próprio

Endereço: Rua Tessália Vieira de Camargo, 126
Bairro: Barão Geraldo **CEP:** 13.083-887
UF: SP **Município:** CAMPINAS
Telefone: (19)3521-8936 **Fax:** (19)3521-7187 **E-mail:** cep@fcm.unicamp.br

Considerações sobre os Termos de apresentação obrigatória:

Foram analisados os seguintes documentos de apresentação obrigatória:

1- Folha de rosto para pesquisas envolvendo seres humanos: Foi apresentado o documento "folhaderostoMarta.pdf" de 10/09/2016 e 08/12/2016.

2- Projeto de pesquisa: Foram analisados os documentos "PB_INFORMAÇÕES_BÁSICAS_DO_PROJETO_790737.pdf" de 29/10/2016 e "PlataformaMarta.pdf" de 08/09/2016 e 08/12/2016.

3- Orçamento financeiro e fontes de financiamento: Informações sobre o orçamento financeiro incluídas nodocumento. Adequado.

4- Cronograma: Informações sobre o cronograma incluídas nos documentos. Adequado.

5- Termo de Consentimento Livre e Esclarecido (TCLE): Foi apresentado o documento "TCLEMarta.pdf" de08/09/2016 e 08/12/2016. Adequado.

6- Currículo do pesquisador responsável e demais colaboradores: O currículo da pesquisa. Adequado.

7– Outros documentos que acompanham o Protocolo de Pesquisa:

- Carta de autorização para coleta de dados assinada pelo Secretário Municipal de Saúde de Campinas.

Documento "aceitedoprojeto.pdf" de 29/10/2016

- Comprovante de vínculo com a instituição foi apresentada a carteira funcional. Documento "RA.pdf" de08/09/2016.

Recomendações:
Nenhuma.

Conclusões ou Pendências e Lista de Inadequações:
Todas as pendências apontadas foram esclarecidas.

TERMO DE CONSENTIMENTO LIVRE E ESCLARECIDO

1. No quarto parágrafo que trata dos procedimentos que envolvem a participação no estudo:

a) Lê-se que a entrevista terá "duração variável, mas controlada por você mesmo". Mesmo estando escritoque a duração da entrevista será variável, a pesquisadora deveria determinar aproximadamente quanto tempo à pessoa demorará em responder a entrevista. Essa informação é necessária que a pessoa possa decidir se tem ou não condições de despender esse tempo para a entrevista.

b) Nesse parágrafo deveria estar escrito que "As entrevistas serão realizadas em local de sua escolha".RESPOSTA: No Termo foi adicionado a seguinte informação " em local de sua escolha, com duração aproximada de 30 minutos".

ANÁLISE: PENDÊNCIA ATENDIDA

c) Descrever como as entrevistas serão armazenadas, por quanto tempo e como será feito o seu descarte. RESPOSTA: Nesse documento lê-se que "... será mantida em sigilo de qualquer pessoa que não faça parte da equipe desta pesquisa. Sua identidade não será divulgada em nenhuma fase da pesquisa ou na divulgação dos resultados da mesma. Os dados colhidos serão armazenados em arquivos digitais, protegidos por senha, aos quais só a pesquisadora e orientadora terão acesso. Serão armazenados durante o desenvolvimento do estudo e destruídos após 60 meses".

**Endereço:**	Rua Tessália Vieira de Camargo, 126				
**Bairro:**	Barão Geraldo		**CEP:**	13.083-887	
**UF:** SP		**Município:**	CAMPINAS		
**Telefone:**	(19)3521-8936	**Fax:**	(19)3521-7187	**E-mail:**	cep@fcm.unicamp.br

2. No item que se refere ao "Sigilo e privacidade":
a) Consta que "não sendo divulgado seu nome em nenhuma fase da pesquisa". Há a necessidade de especificar que na divulgação dos resultados do estudo, o nome da pessoa também não será mencionado. b) Também deve estar descrito que nenhuma informação será dada a outras pessoas que não façam parte da equipe de pesquisa.

ANÁLISE: PENDÊNCIA ATENDIDA

3. No item referente ao "Ressarcimento das despesas" está escrito que "Você não receberá qualquer valorem dinheiro pela sua participação, assim como não haverá qualquer despesa decorrente dessa participação". Deve ser esclarecido que não haverá ressarcimento das despesas porque o participante não terá nenhum tipo de gasto já que a pesquisadora irá se deslocar até os participantes par realizar a entrevista.

RESPOSTA: Consta que "... não haverá qualquer despesa decorrente dessa participação, visto que a

pesquisadora irá se deslocar até o participante para realizar a pesquisa".
ANÁLISE: PENDÊNCIA ATENDIDA

3. No parágrafo que faz referência aos "Riscos e/ou desconfortos" envolvidos na pesquisa, há alguns itensque precisam de esclarecimento:

a) Lê-se que pode haver um "desconforto com o tempo dispendido ao longo da entrevista e/ou oficina", noentanto nesse mesmo documento no item que descreve no que consiste a participação da pessoa fala-se somente em entrevista e não em oficina. As informações devem ser consistentes.

b) Também está escrito que "a pesquisadora compromete a minimizar esta ocorrência", explicar como apesquisadora pretende minimizar essa ocorrência.

RESPOSTA: Esse item foi reescrito da seguinte forma "Esta pesquisa não apresenta riscos previsíveis, mas poderá haver desconforto com o tempo dispendido ao longo da entrevista, sendo que a pesquisadora compromete a minimizar esta ocorrência lhe deixando à vontade para terminar a entrevista quando você julgar necessário".
ANÁLISE: PENDÊNCIAS ATENDIDAS

5. Indenização: A Resolução 466/12 (item IV.3) estabelece que deve estar obrigatoriamente contido no TCLE "h) explicitação da garantia de indenização diante de eventuais danos decorrentes da pesquisa". Também determina que "os participantes da pesquisa que vierem a sofrer qualquer tipo de dano resultante de sua participação na pesquisa, previsto ou não no TCLE, têm direito à indenização, por parte do pesquisador, patrocinador e das instituições envolvidas" (item V.7). Cabe enfatizar que a questão da indenização não é prerrogativa da Resolução 466/12, estando prevista no código civil. Portanto, solicitamos que seja assegurado, de forma clara e afirmativa, que o participante de pesquisa tem direito à indenização em casos de danos decorrentes da pesquisa.

RESPOSTA: O seguinte texto foi acrescentado no Termo "Entretanto, você terá a garantia ao direito a indenização diante de eventuais danos decorrentes de sua participação na pesquisa, previsto ou não neste TCLE, por parte do pesquisador e instituições envolvidas. Cabe enfatizar que o direito a indenização está prevista pelo Código Civil".
ANÁLISE: PENDÊNCIA ATENDIDA

UNICAMP - FACULDADE DE CIÊNCIAS MÉDICAS DA UNIVERSIDADE DE CAMPINAS

6. Há a necessidade de acrescentar um parágrafo que descreve a função do “Comitê de Ética em

Pesquisa (CEP)”. No site do CEP tem uma estrutura básica de como deve ser escrito o TCLE que tem esse item. Esse documento pode ser acessado através do link https://www.prp.unicamp.br/pt-br/projeto-depesquisa e clique em cima do item “3. Estrutura básica para Termo de Consentimento Livre e Esclarecido (TCLE)”

RESPOSTA: O parágrafo foi acrescentado.

ANÁLISE: PENDÊNCIA ATENDIDA

7. No item “Consentimento livre e esclarecido:” tem algumas informações que devem ser acrescentadascomo a pessoa deve declarar que receberá uma via do TCLE assinada e rubricada por ela e pela pesquisadora. No site do CEP, no documento com a estrutura básica do TCLE, consta as informações que devem constar nesse item. Esse documento pode ser acessado através do link citado acima.

RESPOSTA: Foi adicionado “...participar e declaro estar recebendo uma via original deste documento assinada pelo pesquisador e por mim, estando todas as folhas rubricadas por nós”.

ANÁLISE: PENDÊNCIA ATENDIDA

**Endereço:** Rua Tessália Vieira de Camargo, 126			
**Bairro:** Barão Geraldo		**CEP:** 13.083-887	
**UF:** SP	**Município:** CAMPINAS		
**Telefone:** (19)3521-8936	**Fax:** (19)3521-7187	**E-mail:** cep@fcm.unicamp.br	

8. a) Inserir os campos para serem colocadas as rubricas do “participante da pesquisa” e do “pesquisador”nas páginas do TCLE. b) Deve ser inserido a numeração das páginas do TCLE, de forma a indicar, também, o número total de páginas como por exemplo: 1 de 2.

RESPOSTA: As alterações solicitadas foram realizadas.

ANÁLISE: PENDÊNCIA ATENDIDA

9. Um dos critérios de exclusão da pesquisa é a pessoa não aceitar que a entrevista seja gravada segundoconsta no documento “Informações sobre a pesquisa”. Essa informação deve estar claramente descrita no TCLE. Da forma como essa informação está inserida no TCLE parece que a pessoa pode optar por aceitar ou não gravar a entrevista e independentemente disso irá participar da pesquisa.

RESPOSTA: A pesquisadora acrescentou no item “Procedimentos”, “ ... esclarecendo que, caso você não autorize a gravação, será excluído do estudo: (_) autorizo que minha entrevista seja gravada; ( ) não autorizo que a minha entrevista seja gravada.

ANÁLISE: PENDÊNCIA ATENDIDA

**Endereço:**	Rua Tessália Vieira de Camargo, 126				
**Bairro:**	Barão Geraldo		**CEP:**	13.083-887	
**UF:** SP	**Município:**	CAMPINAS			
**Telefone:**	(19)3521-8936	**Fax:**	(19)3521-7187	**E-mail:**	cep@fcm.unicamp.br

INFORMAÇÕES BÁSICAS DO PROJETO E PROJETO DE PESQUISA

- Cronograma: deve ser revisto já que o início da pesquisa está previsto para o dia 1 de novembro de 2016. Observar que o início do estudo somente poderá ser realizado após aprovação pelo CEP.

RESPOSTA: O cronograma da pesquisa foi alterado, sendo que o início da coleta de dados acontecerá em janeiro de 2017.

ANÁLISE: PENDÊNCIA ATENDIDA

Considerações Finais a critério do CEP:

- O sujeito de pesquisa deve receber uma via do Termo de Consentimento Livre e Esclarecido, na íntegra,por ele assinado (quando aplicável).

- O sujeito da pesquisa tem a liberdade de recusar-se a participar ou de retirar seu consentimento emqualquer fase da pesquisa, sem penalização alguma e sem prejuízo ao seu cuidado (quando aplicável).

- O pesquisador deve desenvolver a pesquisa conforme delineada no protocolo aprovado. Se o pesquisadorconsiderar a descontinuação do estudo, esta deve ser justificada e somente ser realizada após análise das razões da descontinuidade pelo CEP que o aprovou. O pesquisador deve aguardar o parecer do CEP quanto à descontinuação, exceto quando perceber risco ou dano não previsto ao sujeito participante ou quando constatar a superioridade de uma estratégia diagnóstica ou terapêutica oferecida a um dos grupos da pesquisa, isto é, somente em caso de necessidade de ação imediata com intuito de proteger os participantes.

- O CEP deve ser informado de todos os efeitos adversos ou fatos relevantes que alterem o curso normal doestudo. É papel do pesquisador assegurar medidas imediatas adequadas frente a evento adverso grave ocorrido (mesmo que tenha sido em outro centro) e enviar notificação ao CEP e à Agência Nacional de Vigilância Sanitária – ANVISA – junto com seu posicionamento.

- Eventuais modificações ou emendas ao protocolo devem ser apresentadas ao CEP de forma clara esucinta, identificando a parte do protocolo a ser modificada e suas justificativas e aguardando a aprovação do CEP para continuidade da pesquisa. Em caso de projetos do Grupo I ou II apresentados anteriormente à ANVISA, o pesquisador ou patrocinador deve enviá-las também à mesma, junto com o parecer aprovatório do CEP, para serem juntadas ao protocolo inicial.

- Relatórios parciais e final devem ser apresentados ao CEP, inicialmente seis meses após a data desteparecer de aprovação e ao término do estudo.

- Lembramos que segundo a Resolução 466/2012 , item XI.2 letra e, “cabe ao pesquisador apresentar dados solicitados pelo CEP ou pela CONEP a qualquer momento”.

Endereço: Rua Tessália Vieira de Camargo, 126
Bairro: Barão Geraldo **CEP:** 13.083-887
UF: SP **Município:** CAMPINAS
Telefone: (19)3521-8936 **Fax:** (19)3521-7187 **E-mail:** cep@fcm.unicamp.br

UNICAMP - FACULDADE DE CIÊNCIAS MÉDICAS DA UNIVERSIDADE DE CAMPINAS

Continuação do Parecer: 1.885.225

Este parecer foi elaborado baseado nos documentos abaixo relacionados:

Tipo Documento	Arquivo	Postagem	Autor	Situação
Informações Básicas do Projeto	PB_INFORMAÇÕES_BÁSICAS_DO_PROJETO_790737.pdf	08/12/2016 11:54:20		Aceito
Outros	Cartaresposta.pdf	08/12/2016 11:41:35	MARTA PATRICIA SPAZAPAN	Aceito
TCLE / Termos de Assentimento / Justificativa de Ausência	TCLE2.pdf	08/12/2016 11:37:40	MARTA PATRICIA SPAZAPAN	Aceito
Outros	aceitedoprojeto.pdf	29/10/2016 13:43:02	MARTA PATRICIA SPAZAPAN	Aceito
Folha de Rosto	folhaderostoMarta.pdf	10/09/2016 00:30:26	MARTA PATRICIA SPAZAPAN	Aceito
Outros	RA.pdf	08/09/2016 21:34:57	MARTA PATRICIA SPAZAPAN	Aceito
Projeto Detalhado / Brochura Investigador	PlataformaMarta.pdf	08/09/2016 21:31:51	MARTA PATRICIA SPAZAPAN	Aceito

Situação do Parecer:

Aprovado

Necessita Apreciação da CONEP:

Não

CAMPINAS, 04 de Janeiro de 2017

Assinado por:
Renata Maria dos Santos Celeghini
(Coordenador)

Endereço: Rua Tessália Vieira de Camargo, 126
Bairro: Barão Geraldo **CEP:** 13.083-887
UF: SP **Município:** CAMPINAS
Telefone: (19)3521-8936 **Fax:** (19)3521-7187 **E-mail:** cep@fcm.unicamp.br
